# Efficacy and safety of minocycline in retinitis pigmentosa: a prospective, open-label, single-arm trial

**DOI:** 10.1038/s41392-024-02037-2

**Published:** 2024-12-04

**Authors:** Yuxi Chen, Yuan Pan, Yanyan Xie, Yuxun Shi, Yao Lu, Yiwen Xia, Wenru Su, Xiaoqing Chen, Zuoyi Li, Minzhen Wang, Siyu Miao, Yating Yang, Chenjin Jin, Guangwei Luo, Shixian Long, Hui Xiao, Chuangxin Huang, Jian Zhang, Dan Liang

**Affiliations:** Department of Ocular Immunology, State Key Laboratory of Ophthalmology, Zhongshan Ophthalmic Center, Sun Yat-sen University, Guangdong Provincial Key Laboratory of Ophthalmology and Visual Science, Guangdong Provincial Clinical Research Center for Ocular Diseases, Guangzhou, China

**Keywords:** Immunotherapy, Neurodevelopmental disorders, Cell death in the nervous system, Neuroimmunology

## Abstract

Retinitis pigmentosa (RP) is characterized by progressive photoreceptor cells death accelerated by the proliferation and activation of microglia pathologically. No consensus exists on the treatment. Minocycline is recognized as a microglia inhibitor with great anti-inflammatory and neuro-protective functions. However, efficacy of minocycline in RP patients is lacking. We conducted a prospective, open-label, and single-arm trial, in which daily oral minocycline of 100 mg was administered for 12 months in RP patients with light-adapted 30 Hz flicker electroretinography (ERG) amplitude >0 µV in at least one eye (NCT04068207). The primary outcome was the proportion of participants with improvement in the ERG amplitude at month 12. The secondary outcomes included improvements of the following items: other ERGs amplitudes, visual field, best-corrected visual acuity, contrast sensitivity, color vision, and NEI-VFQ-25. 35 of 288 patients met inclusive criteria were enrolled (median [IQR] age, 36 [31–45] years; 17 female [48.6%]). 32 participants completed all examinations, while 3 participants completed the 12-month online visit via conducting NEI-VFQ-25. The primary outcome showed improvement was 34.3% (12 of 35 [95% CI 19.1–52.2]). Similarly, all secondary outcomes showed improvements. Adverse events were reported in 22 participants (62.9%) and were all resolved without extra medication during the study period. No severe adverse events were recorded. Our findings identified daily oral minocycline of 100 mg for 12 months was beneficial in improving the visual function of RP patients with good safety. This study indicates minocycline may be a promising therapy for RP, but a randomized controlled trial is still needed of further exploration.

## Introduction

Retinitis Pigmentosa (RP) is a genetically heterogeneous retinal degenerative disorder with an estimated worldwide prevalence of 1/9000–1/850,^1,2^ acting as a leading cause of visual disability and affecting more than 1.5 million patients all over the world.^3^ It is characterized by the ingravescent degeneration of photoreceptors, retinal pigment epithelium, and other retinal cells, leading to progressively worsening symptoms including nyctalopia, subsequent loss of peripheral vision, and consequent deterioration of central vision, ultimately resulting in legal blindness. The clinical diagnosis of RP is not hard to make by a comprehensive evaluation of characteristic symptoms, fundus abnormalities, examinations of visual function and retinal structure, and related gene mutations. Unfortunately, the treatment for RP is deeply fraught with difficulties and frustration. To our knowledge, there is indeed no consensus on treatments to slow the process of RP till now.^3^

The apoptosis of rods is considered the trigger for the pathologic process of RP, and it has been hypothesized that increased intake of compounds required for rhodopsin synthesis, a light-sensitive protein, might halt the progression of RP.^3^ The efficacy of supplementations, including vitamin A,^4,5^ 9-cis-beta-carotene,^6^ docosahexaenoic acid (DHA),^7^ and N-acetylcysteine (NAC)^8^ have been studied to slow down, stop or even reverse the progression of RP in several clinical trials. However, the results have not shown clear evidence leading to clinical physicians being cautious about these treatments and then limiting their widespread adoption in clinical practice. Invasive strategies, including gene therapies and stem cell therapies, are also gradually explored to treat RP in relatively advanced stage.^3,9^ To date, approximately 90 gene mutations associated with RP have been identified,^10^ while gene therapies are only available for few specific mutations, such as RPE65 and RPGR.^9^ Viral vectors, the necessary vehicle in gene therapies, have relatively limited cargo capacity and are unable to carry large cDNAs of genes involved in RP pathogenesis, such as ABCA4.^11^ Consequently, gene therapies are applicable to a small subset of individuals with RP who possess the specific mutation sites. As for stem cell therapies, immune effects, transplanted cell survival, control of differentiation, and safety issues are still problems needed to be solved.^12^ In addition to these strategies, optogenetic therapy is an emerging invasive approach aiming at completely blind patients. It requires precise expression of light-sensitive proteins, control of light stimulation, and equipment dependence, which still needs further pre-clinical researches.^13^ Due to all these challenges, these invasive therapies are only investigated for patients in the relatively advanced stage of RP until now. So, the key of RP treatment is to explore clinically accessible and financially affordable strategies which could slow disease progression of RP patients.

Microglia, a kind of vital immune cell located in the nervous system, has been shown to play a crucial role in various neurodegenerative diseases including RP.^14^ In various models, such as Alzheimer’s disease and Parkinson’s disease, microglial activation has been observed near the lesions and microglial inhibition could delay disease progression.^15^ In RP animal models, such as P23H rats and rd10 mice, it has been revealed that the proliferation and activation of microglia accelerate the progression of photoreceptor cells death by promoting inflammation.^16^ Minocycline, a second-generation tetracycline antibiotic with an advantage of penetration through the blood-brain barrier,^17^ has been found to suppress the proliferation and activation of microglia, thereby inhibiting inflammation and protecting neurocytes.^18^ In mouse models of RP, minocycline has been found to significantly improve retinal structure and function by inhibiting microglia activation and reducing photoreceptor cells apoptosis.^19^ Recently, our team has also demonstrated that minocycline prevents photoreceptor degeneration in rd1 mice, a classic RP animal model, through modulating mitochondrial homeostasis.^20^ In the aspect of clinical trials, the effect of minocycline on neurodegenerative diseases has been investigated. Several clinical trials have provided evidence to support the consideration of minocycline in neurodegenerative diseases such as multiple sclerosis,^21^ Huntington’s disease^22^, and Parkinson’s disease.^23^ Recently, Dave’s team reported that oral minocycline, at a dosage of 100 mg twice per day for 12 months, reduced macular center thickness in 5 RP-associated cystoid macular edema patients.^24^ Meanwhile, in these trials, minocycline was reported to be well tolerated without severe adverse events.

As mentioned above, the progressive neurodegenerative characteristic of RP and the lack of consensus on treatments to slow the process make it urgent to explore possible therapeutic strategies for RP. Growing evidence has shown that minocycline could protect neurons from apoptosis by inhibiting the proliferation and activation of microglia.^18^ Also, the tolerability of minocycline has been demonstrated.^21^ As a result, we designed this single-arm trial to preliminarily investigate the therapeutic potential of minocycline in RP patients. In this trial, we chose electroretinography (ERG) as the primary outcome measure due to its recognized role as a reliable and objective measurement to reflect the abnormalities of photoreceptors. It can provide a quantitative assessment of the severity and monitor the progression of RP, thereby mitigating potential biases associated with single-arm design to the greatest extent. This prospective, open-label, single-arm trial aims to pave a new, cost-effective, and accessible therapeutic way for RP patients desperately seeking for brightness.

## Results

**Fig. 1:**
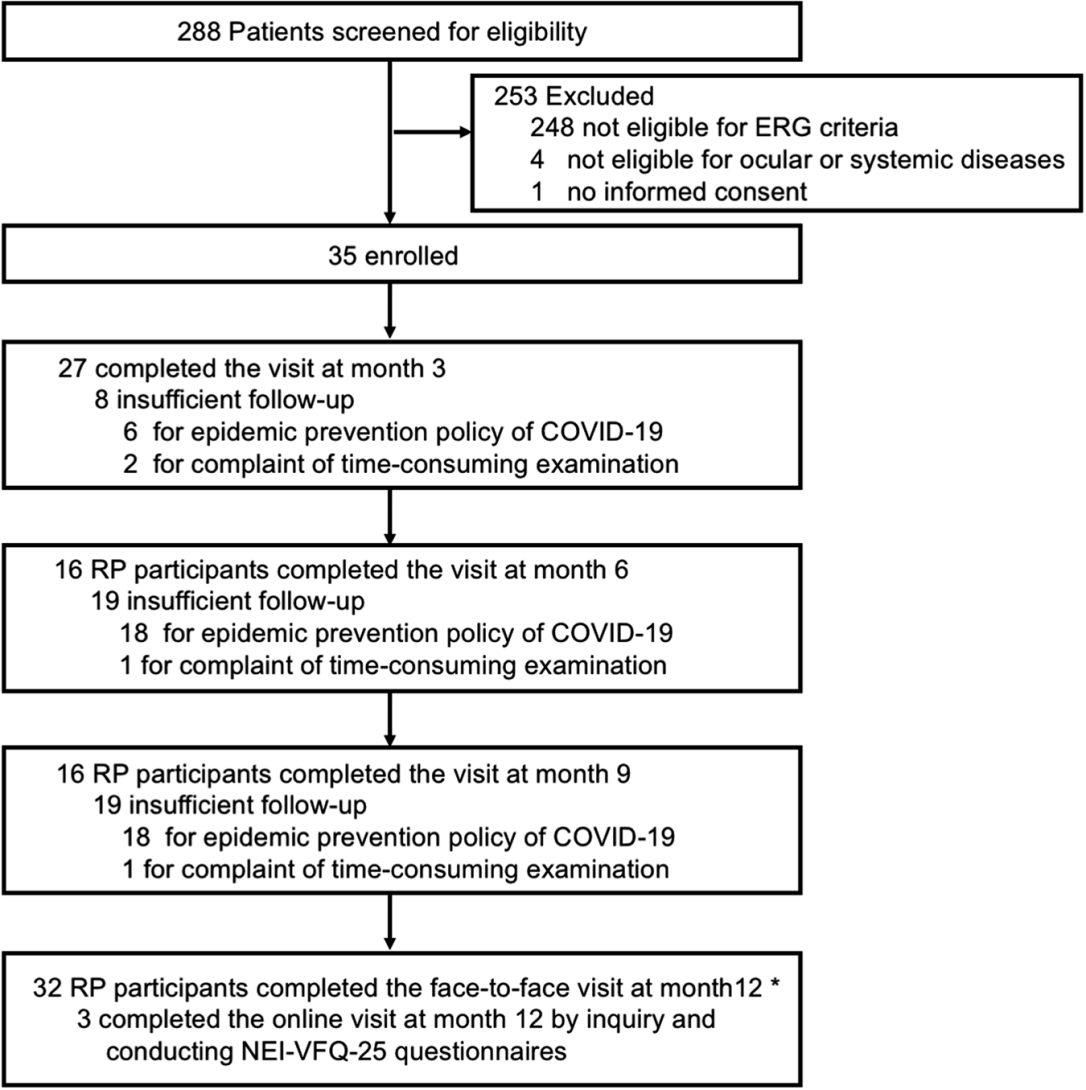
Enrollment and follow-up of Study Patients. *Of these thirty-five participants, thirty-two participants completed all examinations at month 12 by face-to-face visit, while three participants completed the 12-month online visit via inquiry and conducting NEI-VFQ-25 questionnaire without examination of visual function

288 RP patients in China were screened for the trial from August 2019 to December 2021 (Fig. 1). 248 did not meet ERG eligibility criteria, 4 were excluded due to ocular or systemic disease and 1 refused informed consent. A total of 35 participants (18 men and 17 women; median 36 years, IQR 31-45) were enrolled. Among them, 32 participants completed all examinations at month 12 by face-to-face visit, while 3 participants completed the 12-month online visit via conducting NEI-VFQ-25 questionnaires. The median age of disease onset was 31 years old (IQR 25–40) and the median duration of RP was 36 months (IQR 12–60). Nyctalopia appeared to be the most common complaint, accounting for 71% (25/35) of cases. All eyes showed characteristic decreased ellipsoid zone width and reduced ERG response. The baseline of light-adapted 30 Hz flicker ERG amplitude was 36.8µV in the right eye (SD 22.2) and 32.8 µV in the left eye (SD 29.2). MD in visual field at baseline was −21.2 dB in right eye (SD 8.6) and −21.3 dB in the left eye (SD 8.9). Decreased visual acuity happened in 55 of 70 eyes (78.6%) with 0.2 logMAR in the right eye (IQR 0–0.3) and 0.2 logMAR in the left eye (IQR 0-1.0). For the total of 70 eyes, 22 eyes (31.4%) had bone spicule-like pigment formation, and 36 eyes (51.4%) had vascular leakage. 27 out of 35 participants found definite gene mutations involving 19 mutated genes. Detailed baseline characteristics, ocular features, variables, and gene mutations were summarized in Table 1 and Supplementary Tables 1–2. Due to the influence of COVID-19, eleven participants returned for the last visit after month 12 (median 14.2 months, IQR, 13.8-14.9) with continuous drug intake.

**Table 1:** Baseline Demographic Characteristics and Disease Features of Patients

Characteristics	All patients (*N* = 35)
Age at enrollment, median (IQR), y	36 (31–45)
Gender, no. (%)	
Male	18 (51.4)
Female	17 (48.6)
Age at disease onset, median (IQR), y	31 (25–40)
Duration of disease, median (IQR), mo	36 (12–60)
Chief complaint, no. of patients (%)	
Blurred vision	24 (68.6)
Nyctalopia	25 (71.4)
Loss of visual field	22 (62.9)
Clinical Characteristics, no. of eyes (%)	70
Decreased logMAR visual acuity^a^	55 (78.6)
Cataract	8 (11.4)
Vitreous opacification	22 (31.4)
Optic nerve waxy pallor	24 (34.3)
Bone spicule-like pigment formation	22 (31.4)
Decreased ellipsoid zone width	70 (100.0)
Vascular leakage	36 (51.4)
Reduced ERG response	70 (100.0)
Decreased vision field^b^	70 (100.0)
Decreased contract sensitivity^c^	48 (68.6)
Abnormal color vision^d^	56 (80.0)

*IQR* interquartile range, *y* year, *mo* month, *logMAR* logarithm of the minimum angle of resolution

^a^Decreased logMAR visual acuity was defined as logMAR visual acuity higher than zero

^b^Decreased vision field was defined as a mean deviation less than −2 dB

^c^Decreased contract sensitivity was defined as a poor contract sensitivity which was lower than 'Contract Sensitivity Function'

^d^Abnormal color vision was defined as a decrease more than 100 in error score of FM-100 color vision test by the same trainer

### Efficacy outcomes

The primary outcome was 34.3% (12 of 35, 95% CI, 19.1%–52.2%) of participants getting improvement in light-adapted 30 Hz flicker ERG amplitude at month 12 (Table 2).

**Table 2:** Twelve-month treatment outcomes: percentage of improvement from baseline

Outcomes	All patients (*N* = 35) No. (%[95% CI])
Primary outcome	
LA 30 Hz flicker ERG amplitude: % improvement from baseline ≥10%^a^	12 (34.3 [19.1––52.2])
Secondary outcomes	
DA 0.01 ERG b-wave amplitude: % improvement from baseline ≥10%	6 (17.1 [6.6–33.7])
DA 3.0 ERG a-wave amplitude: % improvement from baseline ≥10%	10 (28.6 [14.6–46.3])
DA 3.0 ERG b-wave amplitude: % improvement from baseline ≥10%	12 (34.3 [19.1–52.2])
LA 3.0 ERG a-wave amplitude: % improvement from baseline ≥10%	18 (51.4 [34.0–68.6])
LA 3.0 ERG b-wave amplitude: % improvement from baseline ≥10%	10 (28.6 [14.6–46.3])
DA 3.0 OPS total amplitude: % improvement from baseline ≥10%	8 (22.9 [10.4–40.1])
DA 3.0 OP1 amplitude: % improvement from baseline ≥10%	8 (22.9 [10.4–40.1])
DA 3.0 OP2 amplitude: % improvement from baseline ≥10%	8 (22.9 [10.4–40.1])
DA 3.0 OP3 amplitude: % improvement from baseline ≥10%	11 (31.4 [16.9–49.3])
DA 3.0 OP4 amplitude: % improvement from baseline ≥10%	12 (34.3 [19.1–52.2])
Visual Field MD: % improvement from baseline >0^b^	16 (45.7 [28.8–63.4])
BCVA: % improvement from baseline >0	12 (34.3 [19.1–52.2])
CS (1.5cpd): % improvement from baseline >0	10 (28.6 [14.6–46.3])
CS (3cpd): % improvement from baseline >0	16 (45.7 [28.8–63.4])
CS (6cpd): % improvement from baseline >0	16 (45.7 [28.8–63.4])
CS (12cpd): % improvement from baseline >0	16 (45.7 [28.8–63.4])
CS (18cpd): % improvement from baseline >0	14 (40.0 [23.9–57.9])
Color Vision: % improvement from baseline >0	17 (48.6 [31.4–66.0])
NEI-VFQ-25 Score: % improvement from baseline >0	30 (85.7 [69.7–95.2])

*CI* confidence interval, *LA* light-adapted, *DA* dark-adapted, *ERG* electroretinogram, *OP* oscillatory potential, *MD* mean deviation, *BCVA* best corrected visual acuity, *CS* contrast sensitivity, *NEI-VFQ-25* National Eye Institute Visual Functioning Questionnaire 25

^a^The improvement in ERG amplitudes was defined as a change from baseline with at least 10% increase in any eye

^b^The improvement in other secondary outcomes except ERG amplitudes was defined as a change from baseline over zero in any eye

For the secondary outcomes of other ERG indexes, 6 participants (17.1%, 95% CI, 6.6%–33.7%) showed improvement in dark-adapted 0.01 ERG b-wave amplitudes. The proportions of improvement in dark-adapted 3.0 ERG a- and b-wave amplitudes were 28.6% (95%CI, 14.6%–46.3%) and 34.3% (95%CI, 19.1%–52.2%), respectively. For amplitudes of light-adapted 3.0 ERG, participants who got improved in a- and b-wave amplitudes were 18 (51.4%, 95% CI, 34.0%–68.6%) and 10 (28.6%, 14.6%–46.3%), respectively. As for the amplitudes of total oscillatory potentials (OPs) as well as OP1 to OP4, the improvement rates ranged from 22.9% (95%CI, 10.4%–40.1%) to 34.3% (95%CI, 19.1%–52.2%) (Table 2).

Furthermore, 16 participants (45.7%, 95% CI, 28.8%–63.4%) got improvement in MD of visual field. 12 participants (34.3%, 95% CI, 19.1%–52.2%) showed improvement in BCVA, and contrast sensitivity at spatial frequencies of 1.5, 3, 6, 12, and 18 cpd also showed improvement to varying degrees. 17 participants (48.6%, 95% CI, 31.4%–66.0%) showed improvement in color vision (Table 2).

In addition, we carried out a detailed questionnaire to evaluate the vision-related quality of life in NEI-VFQ-25 of these participants and observed that 30 participants (85.7%, 95% CI, 69.7%–95.2%) got improvement (Table 2).

### Safety outcomes

Of the 35 enrolled participants, 22 participants (62.9%) reported a total of 58 adverse events with no severe adverse events. No other adverse events on lab tests (including function of liver, kidney, and thyroid) as well as systemic condition were recorded. The most common adverse events were dermatologic symptoms reported by 17 participants (48.6%). Additionally, 10 participants (28.6%) reported gastrointestinal symptoms and 6 participants (17.1%) reported dizziness as a neurologic symptom during this 12-month trial. All events were resolved during the study period without requiring extra medication and no participant withdrew from this trial due to adverse events (Table 3).

**Table 3:** Twelve-month adverse events of minocycline for RP patients

Events	All patients (*N* = 35)
Adverse events^a^	22 (62.9)
Severe adverse events	0
General	8 (22.9)
Fatigue	3 (8.6)
Memory Loss	4 (11.4)
Tinnitus	1 (2.9)
Weight Gain	1 (2.9)
Dermatologic symptoms	17 (48.6)
Pruritus	10 (28.6)
Skin acne	3 (8.6)
Hyperpigmentation	11 (31.4)
Photosensitivity	1 (2.9)
Gastrointestinal symptoms	10 (28.6)
Nausea	4 (11.4)
Vomiting	2 (5.7)
Abdominal pain	1 (2.9)
Abdominal distension	2 (5.7)
Constipation	2 (5.7)
Diarrhea	3 (8.6)
Neurologic symptoms	6(17.1)
Dizziness	6 (17.1)
Headache	1 (2.9)
Infections	3 (8.6)
Vaginitis	3 (8.6)

Values are reported as No. (%)

^a^Multiple adverse events may occur in the same participant. All events were resolved during the study period without requiring extra medication

## Discussion

This prospective, open-label, single-arm trial showed that 12-month treatment of oral minocycline 100 mg per day was beneficial for RP participants with good tolerability. We observed that 34.3% (95% CI, 19.1%–52.2%) participants had an improvement of light-adapted 30 Hz flicker ERG amplitude objectively. Consistently, the results also showed improvement rates of other ERG indexes ranging from 17.1% to 51.4%. Additionally, visual functions including visual field, BCVA, contrast sensitivity, color vision, and NEI-VFQ-25 were improved. The safety profile in this study was well accepted. For RP is a gradually worsening disease without admitted therapies to improve the visual function in patients, these findings suggest that minocycline may be a promising therapy for RP patients with great safety.

RP is a neurodegenerative disease, in which the proliferation and activation of microglia play a role in neuron apoptosis as it does in other neurodegenerative diseases.^25^ In retinal inflammation-mediated degenerative diseases, microglial proliferation and activation could be partly regulated by PI3K/AKT/NF-κB pathway, thus resulting in retinal neuron apoptosis.^26^ A study claimed that the activation process was regulated by CX3CL1-CX3CR1 signaling pathway leading to the secretion of inflammatory cytokines such as IL-6 and TNF-α and further promoting the apoptotic cascades of photoreceptors in RP, indicating that microglial activation was a critical factor of photoreceptor apoptosis in RP.^27^ Also, minocycline has been reported to exert anti-inflammatory effects by inhibiting CX3CR1-positive microglial activation in RP.^19^ Additionally, minocycline could inhibit microglial activation through PI3K/AKT/NF-κB pathway.^28^ The efficacy and safety profile of minocycline has also been investigated on neurodegenerative diseases in clinical trials. For instance, the New England Journal of Medicine has reported a RCT in 2017 claiming that minocycline could significantly reduce the risk of conversion from clinically isolated syndrome to multiple sclerosis compared with placebo.^21^ Other trials have also provided evidence to support the consideration of minocycline in treating Huntington’s disease^22^ and Parkinson’s disease.^23^ Together, these findings have provided convincing evidence in terms of mechanism and efficacy of minocycline for us to perform this study.

As we all know, light-adapted 30 Hz flicker ERG amplitude is largely generated by cone cells and reflects cone function.^29^ In our trial, we chose it as the primary outcome for the reason that it has been regarded as the most sensitive and stable ERG marker for RP.^30^ A majority of previous studies on natural decay of RP and assessment of treatment efficacy were all based on this index.^31^ So, we enrolled RP patients with this index >0 µV in at least one eye. To further rigorously evaluate the efficacy of minocycline on RP, an increase not less than 10% compared with the baseline was set as an improvement in this trial. The results showed that 34.3% participants (12/35, 95% CI, 19.1%–52.2%) got improved. According to previous studies, an 7.1%–14.6% annual decay of light-adapted 30 Hz flicker ERG amplitude exists.^32,33^ Considering the progressive course of RP, an increase of this index by not less than 10% compared with the baseline is clinically encouraging and significant. Taken together, we provided a new approach for RP patients to apply minocycline, which may increase the survival of photoreceptors.

Other ERG indexes, including amplitudes of dark-adapted 0.01 and 3.0 ERG, light-adapted 3.0 ERG, and OPs were regarded as second outcomes in this trial, and they were also used as outcomes reflecting retinal function in retina diseases, including diabetic retinopathy and familial exudative vitreoretinopathy.^34,35^ Although dark-adapted 0.01 ERG amplitude was hardly detectable at enrollment, we still found that 16 participants had detectable 0.01 ERG b-wave amplitude, and 6 participants (6/16, 37.5%) got improvement, which reflects an increase in function of rods. Dark-adapted 3.0 ERG is a mixed rod and cone response, and improvement rates of a- and b- wave amplitudes were detected in 28.6% and 34.3% of participants in this trial, respectively. A- and b- wave amplitudes of light-adapted 3.0 ERG, representing the cone responses, were also improved in 34.3% and 51.4% of participants. OPs appear to reflect the inner layer of retina, and the amplitudes also improved with different degrees in this trial.^31^ These secondary outcomes showed great consistency with the primary outcome.

Progressive death of rods gradually causes loss of peripheral and middle visual field at the relatively early stage. MD of visual field is a common parameter used to evaluate the development of retinal diseases, including RP and glaucoma.^36^ A decay of MD in visual field ranging from 5.6% to 20.1% per year in RP with different mutations has been reported.^37,38^ Excitingly, 45.7% (16/35) participants got improvement in our trial.

BCVA is the most intuitive representation of cone function. Additionally, contrast sensitivity appears to be much more related with vision-related activities and visual function damage.^39^ We observed that 34.3% of participants showed improvement in BCVA, and different improvement rates of all LogCSs ranged from 28.6% to 45.7%. In comparison to the above two variables, color vision starts with the absorption of light in cones of better sensitivity, and we found that 17 participants (48.6%) had decreased error scores after treatment. All these results showed a promising efficacy of minocycline for cone cells in RP.

In addition, NEI-VFQ-25 is employed to measure the vision-related quality of life of patients in clinical trials^40^ and our results showed 85.7% of participants obtained higher scores after receiving 12 months of minocycline.

Vitamin A, 9-cis-beta-carotene, NAC, and DHA, as pharmacological approaches, were investigated in the treatment of RP in previous studies.^4,6–8^ While no consensus on whether which approach could slow the process of RP was reached in clinic. Differences among these studies should be noticed when interpreting the results. Each study has their inclusion criteria enrolling patients, time window for intervention, and outcome measurements, it is not available to compare improvement of the visual parameters between our result with those studies, even among those studies.

Previous studies have indicated that about 60%–70% of RP patients possess identifiable gene mutations,^3^ while there are 88 detectable gene mutations related to the onset of RP.^10^ In our study, we found that 27 of 35 RP participants (77%) had definite gene mutation involving 19 genes, which was in consistent of previous studies. To be more specific, the mutation of USH2A was detected in 5 participants, and the rest of 18 genes including PDE6B, RPILI were detected in no more than 2 participants. The result demonstrated a dispersed nature mutation type which was consistent with published article. So it is not available to establish a link between the outcome of the treatment and the type of mutations with a relatively small sample size in this study. Furthermore, all the participants were Asian, which may limit the reach of our findings to other races.

Regarding the safety of oral minocycline 100 mg daily for 12 months, our trial recorded a total of 58 adverse events occurring in 22 participants (62.9%). The most common adverse event was dermatologic symptoms reported by 17 patients (48.6%), and gastrointestinal symptoms occurred in 10 participants (28.6%). No severe adverse event was reported. After adjusting the timing of minocycline intake to after dinner and avoiding extra sun exposure, participants who experienced gastrointestinal and dermatologic symptoms all resolved within 3 months without any medication. In previous studies of 200 mg minocycline, gastrointestinal symptoms are the most common adverse event ranging from 20 to 44%.^41,42^ A recent study showed that the 3-year treatment of 200 mg minocycline is relatively well tolerated in patients with age-related macular degeneration.^43^ However, the diverse mechanisms of minocycline, an antimicrobial agent, should raise concerns regarding long-term side effects.

In general, our findings revealed that oral minocycline could significantly improve the visual function and vision-related quality of life of RP patients. However, the results need to be interpreted with caution, since the relatively small sample size, absence of control group may produce potential biases and uncontrollable confounding factors, increasing the uncertainty in the interpretation of data on drug efficacy and safety in this single-arm study. But we still want to emphasize that this is a study with a fair scientific standard. Also, we applied an objective variable, ERG, as the primary outcome, to mitigate this potential biases as much as possible. Meanwhile, multiple variables were also applied as secondary outcomes to evaluate the efficacy of minocycline and provide supportive data for our conclusion. We do believe the encouraging findings have significant implications for both doctors and patients for the reason that RP is a progressively worsening disease. Hopefully, these findings will offer a glimmer of hope for the current situation of RP treatment.

In summary, this study does show a potential benefit of minocycline for RP patients due to improved visual function and good safety profile. Additionally, compared with other invasive therapies of RP, minocycline shows irreplaceable superiorities in terms of its low cost and high accessibility. All these supporting evidence, therefore, encourages the continued exploration of minocycline in RP by conducting a randomized controlled trial.

## Materials and methods

### Study design and oversight

This nonrandomized controlled trial was designed to evaluate the efficacy of minocycline in the treatment of RP, with full-field electroretinography (ffERG, shorted as ERG) as the primary efficacy outcome. This trial was conducted for several reasons. Firstly, RP patients often suffer from progressive deterioration of vision and no approved treatments for RP exist to date. Secondly, ERG is widely recognized as the most sensitive and objective measurement for evaluating photoreceptors function. We specifically recruited RP patients with ERG amplitudes more than 0µV, whose ERG responses will continue to decrease without effective intervention. Thirdly, based on the positive effects of minocycline on visual function observed in animal models of RP, we have grounds to believe that minocycline can improve ERG responses in RP patients. The trial was conducted with sufficient statistical power. The trial protocol was reviewed and approved by the Institutional Review Board and Ethics Committee of Zhongshan Ophthalmic Center, Sun Yat-sen University (Ethics approval number: 2019KYPJ032). It was conducted in accordance with the principles of Helsinki Declaration and was registered in ClinicalTrial.gov, NCT04068207. All participants got written informed consent before enrollment. Data were collected by investigators who vouch for the completeness, accuracy of the data, and the fidelity of the trial to the protocol.

### Inclusion and exclusion criteria

Patients were eligible between 18 to 60 years with a confirmed diagnosis of RP based on clinical features, visual functional and ocular structure testing. Minimum light-adapted 30 Hz flicker ERG amplitude required for inclusion should be higher than 0µV at least in one eye.

Patients with other ocular disorders contributing to visual dysfunction or ocular structure damage were excluded. Syndromic retinitis pigmentosa patients, such as those with Usher Syndrome or Bardet-Biedl Syndrome, were also excluded. Besides, participants with severe systemic diseases or allergies to tetracyclines or unable to cooperate with testing should not be enrolled in this study. Details inclusion and exclusion criteria are provided in the trial protocol.

### Procedures

Participants were enrolled by investigators at Zhongshan Ophthalmic Center, Sun Yat-sen University. All participants had a screening visit to determine eligibility before enrollment. Participants were provided with minocycline (100 mg) (Hanhui Pharmaceuticals CO., LTD., China) once a day for consecutive 12 months. During the following treatment period, investigative drug boxes containing 100 minocycline capsules were delivered to each participant every three months. Participants were scheduled to return for follow-up visits at months 3, 6, 9, and 12. A detailed scheme of the procedure is provided in the protocol. Delivery of investigative drugs via SF Express (a convenient and efficient express company in China) was permitted if participants were unable to return due to various reasons. Medication compliance was checked by reminding participants via WeChat (an online message communication service) and counting the remaining capsules. All examinations during follow-ups were conducted using the same instrument and pattern and were performed by the same trained investigator.

Each participant underwent a detailed demographic survey, including age, gender, duration of disease, clinical features, and family history. Essential blood tests were performed to evaluate the systemic condition and eliminate contraindications of tetracycline, including whole blood routine, liver function, kidney function, thyroid function, and infectious items. All participants underwent whole exome sequencing to identify gene mutations. Comprehensive ophthalmological evaluations, including slit lamp examination, fundus multiple images, and visual function assessments, were conducted to provide pre-treatment assessment. Best-corrected visual acuity (BCVA) was measured using the Snellen visual chart. ERG (VerisTM, Electro-Diagnostic Imaging Inc., USA) was performed under both dark-adapted and light-adapted conditions using an International Society for Clinical Electrophysiology of Vision–compliant protocol (UTAS 3000 system; LKC Technologies).^44^ Visual field tests were performed using Humphrey (Carl Zeiss Meditec, Dublin, California, USA) central 30–2 threshold, and the mean deviation (MD) for each test was recorded.^45^ Contrast sensitivity was measured using the Functional Acuity Contrast Test (FACT) (Stereo Optical, Inc., Chicago, IL, USA) with room illumination at 85 cd/m^2^, and the results were converted into log units (logCS) for analysis.^46^ Color vision was evaluated using the Farnsworth-Munsell 100-hue test (FM-100) (Munsell Color Company, Inc, Baltimore, MD, USA) under an illuminance of 100Lux, and error scores were recorded for analysis.^47^ The structure of retina was measured by spectral-domain optical coherence tomography (SD-OCT) (Heidelberg Engineering Inc.).^48^ Fundus fluorescein angiography (FFA) (Heidelberg Engineering Inc.) was also performed on all participants if no contraindication was present. Vision-related quality of life was evaluated using the National Eye Institute Visual Functioning Questionnaire (NEI-VFQ-25).^49^ Detailed procedures were described in the protocol.

### Outcomes

The primary outcome was the proportion of participants with improvement in light-adapted 30 Hz flicker ERG amplitude at month 12. Actually, RP is widely accepted as a progressively worsening retinal disease, an increase more than zero can be evaluated as effective in clinical. To estimate more conservatively, the improvement was defined as a change from baseline with at least 10% increase in any eye.^4,33,38^ The secondary outcomes included the proportions of participants with improvements in the following items at month 12: 1) other ERG amplitudes; 2) MD in visual field test; 3) BCVA; 4) LogCS in each spatial frequencies in contrast sensitivity; 5) error scale in the color vision test; 6) scores of NEI-VFQ-25. The definitions of improvement for other ERG amplitudes in the second outcomes were similar to the primary outcome, defined as a change from baseline at least 10% increase in any eye.

### Adverse events

All adverse events were graded according to the National Cancer Institute Common Terminology Criteria for Adverse Events (NCI CTCAE) version 4.03. Investigators conducted detailed consultations and systemic reviews at each visit, and all health-related issues during the entire study period were recorded. Severe adverse events had been reported immediately. Blood tests were regularly monitored every three months and as necessary.

### Sample size and statistical analysis

In this study, the proportion of participants with an increase of 10% or more in light-adapted 30 Hz flicker ERG amplitude from baseline to month 12 was set as the primary efficacy outcome. We determined a sample size of approximately 33 patients based on an estimation that 30% participants got improvement. This sample size would provide sufficient data to exclude an improvement rate of 15% at the lower boundary of the two-sided 95% confidence interval. Considering an anticipated dropout rate of 10% among participants, we planned for a final sample size of 35 patients.

The characteristics of participants were presented as mean (standard deviation, SD) or median (interquartile range, IQR) for continuous variables, and as counts (percentage) for categorical variables. Descriptive statistics were used to assess the efficacy and safety of the intervention. For some indexes of ERG as secondary outcomes, if the amplitude at baseline was recorded as zero, we changed zero into one to calculate the rate of improvement. The Clapper-Pearson method was used to calculate the 95% CI of the proportion parameters. The analysis of efficacy and safety included all enrolled participants who attended the trial. In the primary and secondary outcomes, participants who missed month 12 data for any reason were calculated as no improvement. The statistical analysis was performed using Stata version 14.0 software (StataCorp).

## Supplementary information

Supplementary materials

Trial protocol

## Data Availability

Researchers can request access to deidentified patient-level data to the corresponding authors (liangdan@gzzoc.com, zhangjian3@mail.sysu.edu.cn) after this paper’s publication, which will need the approval of the institutional ethical committees. Access to the requested data requires the signing of a data access agreement. The raw data related to the primary outcomes are available in the manuscript and Supplementary materials.
