# Supplementary material for: Efficacy and safety of minocycline in retinitis pigmentosa: a prospective, open-label, single-arm trial
Source: Signal Transduct Target Ther. 2024 Dec 4;9:339. doi: 10.1038/s41392-024-02037-2 (PMC11615376; doi:10.1038/s41392-024-02037-2)
Supplement: Supplementary file 1 — Supplementary materials [file 41392_2024_2037_MOESM1_ESM.docx]

Supplementary Materials for

Efficacy and safety of minocycline in retinitis pigmentosa:

a prospective, open-label, single-arm trial

Yuxi Chen^*,1^, Yuan Pan^*,^^1^, Yanyan Xie^*,1^, Yuxun Shi^*,1^, Yao Lu^1^, Yiwen Xia^1^,

Wenru Su^1^, Xiaoqing Chen^1^, Zuoyi Li^1^, Minzhen Wang^1^, Siyu Miao^1^, Yating Yang^1^, Chenjin Jin^1^, Guangwei Luo^1^, Shixian Long^1^, Hui Xiao^1^, Chuangxin Huang^1^,

Jian Zhang^#,1^, Dan Liang^#,1^

Correspondence to: liangdan@gzzoc.com, [zhangjian3@mail.sysu.edu.cn](mailto:zhangjian3@mail.sysu.edu.cn)

**This PDF file includes:**

Figure S1

Tables S1–S2

**Figure S1 Amplitudes of light-adapted 30Hz flicker ERG in both eyes of RP participants at baseline and 12-month**

|  | Baseline | | 12-Month | |
| --- | --- | --- | --- | --- |
|  | OD | OS | OD | OS |
| ID1 |  | |  | |
| ID2 |  | |  | |
| ID3 |  | |  | |
| ID4 |  | |  | |
| ID5 |  | |  | |
| ID6 |  | |  | |
| ID7 |  | |  | |
| ID8 |  | |  | |
| ID9 |  | |  | |
| ID10 |  | |  | |
| ID11 |  | |  | |
| ID12 |  | |  | |
| ID13 |  | |  | |
|  |  | |  | |
| ID14 |  | |  | |
| ID15 |  | |  | |
| ID16 |  | |  | |
| ID17 |  | |  | |
| ID18 |  | |  | |
| ID19 |  | |  | |
| ID20 |  | |  | |
| ID21 |  | |  | |
| ID22 |  | |  | |
| ID23 |  | |  | |
| ID24 |  | |  | |
| ID25 |  | |  | |
| ID26 |  | |  | |
| ID27 |  | |  | |
| ID28 |  | |  | |
|  |  | |  | |
| ID29 |  | |  | |
| ID30 |  | |  | |
| ID31 |  | |  | |
| ID32 |  | |  | |
| ID33 |  | |  | |
| ID34 |  | |  | |
| ID35 |  | |  | |

The amplitudes of 30Hz flicker ERG were measured from the trough (N1) to the peak (P1) of the waveform.

* The twelve-month visit of three participants (ID14, ID22, ID31) was completely online.

**Table S1. Baseline of Both Eyes**

| **Variables** | **No.** | **Value** |
| --- | --- | --- |
| Light-adapted 30Hz flicker ERG amplitude, mean (SD), µV |  |  |
| OD | 35 | 36.8 (22.2) |
| OS | 35 | 32.8 (29.2) |
| Dark-adapted 0.01 ERG b-wave amplitude, mean (SD), µV |  |  |
| OD | 16 | 97.7 (67.2) |
| OS | 14 | 84.8 (61.5) |
| Dark-adapted 3.0 ERG a-wave amplitude, mean (SD), µV |  |  |
| OD | 28 | 76.0 (56.8) |
| OS | 26 | 60.5 (52.2) |
| Dark-adapted 3.0 ERG b-wave amplitude, mean (SD), µV |  |  |
| OD | 28 | 132.1 (104.7) |
| OS | 26 | 115.6 (117.9) |
| Light-adapted 3.0 ERG a-wave amplitude, mean (SD), µV |  |  |
| OD | 31 | 23.3 (13.2) |
| OS | 30 | 21.1 (16.5) |
| Light-adapted 3.0 ERG b-wave amplitude, mean (SD), µV |  |  |
| OD | 32 | 55.3 (32.7) |
| OS | 30 | 58.3 (54.4) |
| Dark-adapted 3.0 OPS total amplitude, mean (SD), µV |  |  |
| OD | 14 | 69.8 (36.5) |
| OS | 12 | 96.4 (85.4) |
| Dark-adapted 3.0 OP1 amplitude, mean (SD), µV |  |  |
| OD | 14 | 14.3 (7.1) |
| OS | 11 | 15.5 (12.5) |
| Dark-adapted 3.0 OP2 amplitude, mean (SD), µV |  |  |
| OD | 16 | 30.0 (22.8) |
| OS | 14 | 38.2 (45.0) |
| Dark-adapted 3.0 OP3 amplitude, mean (SD), µV |  |  |
| OD | 13 | 14.1 (5.1) |
| OS | 13 | 14.1 (5.1) |
| Dark-adapted 3.0 OP4 amplitude, mean (SD), µV |  |  |
| OD | 14 | 10.4 (7.0) |
| OS | 12 | 15.0 (13.4) |
| MD in Visual Field, mean (SD), dB |  |  |
| OD | 35 | -21.2 (8.6) |
| OS | 35 | -21.3 (8.7) |
| BCVA, median (IQR), logMAR |  |  |
| OD | 35 | 0.2 (0-0.3) |
| OS | 35 | 0.2 (0-1.0) |
| Contrast Sensitivity 1.5cpd, median (IQR), LogCS |  |  |
| OD | 35 | 1.6 (1.4-1.6) |
| OS | 35 | 1.6 (1.4-1.6) |
| Contrast Sensitivity 3cpd, median (IQR), LogCS |  |  |
| OD | 35 | 1.8 (1.5-1.9) |
| OS | 35 | 1.8 (1.5-1.9) |
| Contrast Sensitivity 6cpd, median (IQR), LogCS |  |  |
| OD | 35 | 1.8 (1.2-2.0) |
| OS | 35 | 1.8 (1.1-2.0) |
| Contrast Sensitivity 12cpd, median (IQR), LogCS |  |  |
| OD | 35 | 1.2 (0.9-1.6) |
| OS | 35 | 1.2 (0.9-1.5) |
| Contrast Sensitivity 18cpd, median (IQR), LogCS |  |  |
| OD | 35 | 0.6 (0.6-1.1) |
| OS | 35 | 0.6 (0.6-1.1) |
| Color Vision, median (IQR) |  |  |
| OD | 33 | 160 (92-276) |
| OS | 33 | 168 (112-276) |
| NEI-VFQ-25 Score, mean (SD) | 35 | 79.1 (10.2) |
| Abbreviations: ERG, eletroretinogram; SD, standard deviation; OD, right eye; OS, left eye; OPS, oscillatory potentials; MD, mean deviation; BCVA, best corrected visual acuity; IQR, interquartile range; logMAR, logarithm of the minimum angle of resolution; cpd: cycle/degree; LogCS, logarithm of contrast sensitivity; NEI-VFQ-25: National Eye Institute Visual Functioning Questionnaire 25. | | |

**Table S2. Individual Participant Data**

| **No.** | **Gender** | **Age (y)** | **Age at disease onset (y)** | **Duration of disease (mo)** | **Gene mutations** | **LA 30Hz flicker ERG amplitude M0 (µV)** | | **LA 30Hz flicker ERG amplitude M12 (µV)** | |
| --- | --- | --- | --- | --- | --- | --- | --- | --- | --- |
|  |  |  |  |  |  | **OD** | **OS** | **OD** | **OS** |
| 1 | F | 41 | 40 | 12 | PRPF4 c.1372G>C | 50.6 | 0.0 | 47.4 | 12.2 |
| 2 | M | 34 | 14 | 240 | TRPM1 c.847G>A:p.Gly283Arg | 34.8 | 20.8 | 38.1 | 26.7 |
| 3 | M | 37 | 33 | 48 | NA | 33.3 | 29.3 | 32.2 | 32.8 |
| 4 | M | 42 | 27 | 180 | NA | 20.0 | 18.9 | 19.6 | 18.5 |
| 5 | F | 33 | 31 | 24 | USH2A c.2802T>G:p.Cys934Trp | 43.0 | 55.9 | 28.9 | 20.1 |
| 6 | M | 36 | 30 | 72 | NA | 54.8 | 22.8 | 42.8 | 49.1 |
| 7 | M | 38 | 33 | 60 | USH2A c.9593A>G:p.Tyr3198Cys | 19.0 | 26.5 | 14.0 | 25.3 |
| 8 | M | 47 | 39 | 96 | NA | 22.4 | 29.5 | 28.1 | 27.2 |
| 9 | M | 40 | 35 | 55 | USH2A c.13339A>G:p.Met4447Val | 43.1 | 28.7 | 16.5 | 16.7 |
| 10 | F | 41 | 40 | 12 | EYS c.7747C>T:p.Arg2583Cys | 28.8 | 14.8 | 23.9 | 10.0 |
| 11 | F | 41 | 40 | 12 | CNGA1 c.1271G>A:p.Arg424Glu | 5.71 | 15.9 | 19.2 | 14.0 |
| 12 | M | 54 | 52 | 24 | RP1L1 c.4030del;p.Thr1344fs | 17.7 | 14.8 | 13.6 | 11.0 |
| 13 | M | 18 | 16 | 24 | PRPF3 c.1481C>T:p.Thr494Met | 57.4 | 58.3 | 56.4 | 29.1 |
| 14 | F | 26 | 25 | 12 | PDE6B c.1117del | 7.9 | 10.1 | - | - |
| 15 | M | 33 | 30 | 36 | NA | 40.2 | 34.4 | 32.9 | 31.3 |
| 16 | M | 23 | 22 | 12 | PROM1 c.279A>G:p.Pro93 | 56.4 | 13.8 | 34.6 | 12.8 |
| 17 | M | 31 | 30 | 12 | IFT140 c.4041-6C>A | 3.5 | 7.3 | 8.7 | 8.9 |
| 18 | F | 33 | 16 | 120 | CRB1 c.716G>C:p.Cys239Ser | 13.8 | 9.6 | 14.1 | 9.3 |
| 19 | F | 31 | 30 | 12 | CTSH c.34G>A:p.A12T | 27.0 | 11.0 | 40.7 | 32.6 |
| 20 | M | 20 | 15 | 60 | RIMS1 c.2770+4C>T | 44.0 | 37.1 | 45.2 | 47.4 |
| 21 | F | 47 | 44 | 36 | RP2 c.127A>G:p.Ser43Gly | 54.8 | 56.3 | 36.7 | 28.6 |
| 22 | M | 22 | 14 | 96 | CHM c.931G>T:p.Glu311* | 35.3 | 36.6 | - | - |
| 23 | F | 58 | 55 | 36 | RP1 c.2168dup:p.Gly724ArgfsTer | 32.2 | 35.8 | 34.6 | 25.1 |
| 24 | F | 31 | 30 | 12 | NA | 52.4 | 20.2 | 33.6 | 26.2 |
| 25 | M | 45 | 41 | 50 | NA | 82.4 | 79.0 | 56.5 | 37.1 |
| 26 | F | 33 | 33 | 2 | LRP5 c.3922G>A:p.Gly1308Ser | 72.7 | 68.3 | 47.1 | 42.2 |
| 27 | M | 45 | 44 | 11 | VCAN c.7111A>T:p.Thr2371Ser | 21.7 | 97.7 | 10.9 | 50.5 |
| 28 | F | 25 | 25 | 6 | MFSD8 c.1066C>T:p.Pro356Ser | 54.6 | 0.0 | 44.1 | 4.5 |
| 29 | F | 28 | 28 | 1 | RP1L1 c.4330G>A:p.Ala1444Thr | 87.1 | 92.9 | 54.2 | 63.5 |
| 30 | M | 48 | 28 | 240 | RIMS1 c.1292A>G:p.Glu431Gly | 72.6 | 123.2 | 70.0 | 140.4 |
| 31 | F | 42 | 40 | 24 | USH2A c.8559-2A>G | 11.5 | 16.7 | - | - |
| 32 | F | 56 | 53 | 36 | PROM1 c.1984A>T:p.Lys662* | 43.2 | 21.2 | 19.1 | 22.5 |
| 33 | F | 46 | 36 | 120 | NA | 15.0 | 18.4 | 10.7 | 13.4 |
| 34 | M | 18 | 13 | 60 | USH2A c.13339A>G:p.Met4447Val | 19.0 | 14.8 | 12.7 | 12.7 |
| 35 | F | 35 | 31 | 48 | PDE6B c.1945A>G:p.Asn649Asp | 11.1 | 7.6 | 0.0 | 0.0 |
| Abbreviations: LA: light-adapted; ERG, eletroretinogram; OD, right eye; OS, left eye; F, female; M, male.  NA: no gene mutation was found by whole exome sequencing.  -: no LA 30Hz flicker amplitude was available in this visit.  Normal limits of LA 30Hz flicker ERG amplitude are 88µV-205µV. | | | | | | | | | |
