# Supplementary material for: Efficacy and safety of minocycline in retinitis pigmentosa: a prospective, open-label, single-arm trial
Source: Signal Transduct Target Ther. 2024 Dec 4;9:339. doi: 10.1038/s41392-024-02037-2 (PMC11615376; doi:10.1038/s41392-024-02037-2)
Supplement: Supplementary file 2 — Trial protocol [file 41392_2024_2037_MOESM2_ESM.pdf]

Protocol

This trial protocol has been provided by the authors to give readers additional information about their work.

Protocol for ——————————————

This supplement contains the following items：

1. Original protocol
2. Final protocol
3. Summary of changes

___________________title________________________

Principal Investigator

Dr. Dan LIANG

Department of Ocular Immunology

Zhongshan Ophthalmic Center, Sun Yat-sen University

54 Xianlie South Road, Guangzhou, China

Tel: (+86)020-87330402

E-mail: liangdan@gzzoc.com

Participating Centers

Zhongshan Ophthalmic Center, Sun Yat-sen University, Guangzhou, Guangdong, China

Chief investigator:

Dr. Dan LIANG

Department of Ocular Immunology

Dr. Yuxi CHEN

Department of Ocular Immunology

Data Manager & Statistician:

Jian ZHANG

Department of Ophthalmology

Grant: Sun Yat-Sen University Clinical Research 5010 Program (2018012).

CLINICAL STUDY PROTOCOL

Minocycline for Retinitis Pigmentosa

Original: 26th January 2019

CONTENTS

1. STUDY PERSONNEL AND CONTACT DETAILS

2. ABBREVATIONS

3. SYNOPSIS

4. STUDY BACKGROUND

5. STUDY PURPOSE AND OBJECTIVES

  5.1. Primary objective

  5.2. Secondary objective

    5.2.1. Key secondary objective

    5.2.2. Other secondary objective

  5.3. Exploratory objective

6. STUDY DESIGN

  6.1. Overview

  6.2. Scheme

  6.3. Participants adherence

7. ADMINISTRATION OF INVESTIGATIVE DRUGS

  7.1. Description

  7.2. Investigative drugs making, packaging and labelling

  7.3. Storage, dispensing and return

8. ELIGIBILITY CRITERIA

  8.1. Inclusion criteria

  8.2. Exclusion criteria

9. STUDY VISIT SCHEDULE AND PROCEDURE

10. OUTCOME MEASURES

  10.1.Primary outcome

10.2.Secondary outcome

11. CLINICAL ASSESSMENT

11.1.General physical examination

11.2.General eye examination

11.3.Lab tests

11.4.Efficacy

11.5.Safety

12. DATA MANAGEMENT

12.1.Raw data forms and entry

12.2.CRF and database

13. MANAGEMENT OF ADVERSE EVENTS

13.1.Intensity of Adverse Events

13.2. Assessing relationship of AEs to investigative drug

13.3. Main adverse events of investigative drug

13.4. Reporting of AEs

13.5. Reporting of SAEs

13.6. Management and follow-up of AEs and SAEs

13.7. Collection of Adverse Event Data

14. STATICAL CONSIDERATION

15. ETHICAL CONSIDERATION

15.1. Ethics committee

15.2. Informed consent

15.3. Participant welfare

15.4. Criteria of discontinuing

1. STUDY PERSONNEL AND CONTACT DETAILS

Principal Investigator

Dr. Dan LIANG

Department of Ocular Immunology

Zhongshan Ophthalmic Center, Sun Yat-sen University

54 Xianlie South Road, Guangzhou, China

Tel: (+86)020-87330402

E-mail: liangdan@gzzoc.com

2. ABBREVATIONS

RP	Retinitis pigmentosa
BCVA	Best-corrected visual acuity
ERG	Electroretinogram
OCT	Optical coherence tomography
VF	Visual field
DHA	Docosahexanoic acid
CME	Cystoid macular edema
AMD	Aged-macular degeneration
DR	Diabetic retinopathy
EZ	ellipsoid zone
NCT	Non-contact tonometer
OPS	Oscillatory Potentials

MD	Mean deviation
PSD	Pattern standard deviation
RBC	red blood cell
WBC	white blood cell
PLT	platelet
Hb	hemoglobin
FT3	free triiodothyronine
FT4	free thyroxine
TSH	thyroid stimulating hormone
ALT	Alanine transaminase
AST	aspartate transaminase
HIV	Human immunodeficiency virus
HBV	hepatitis B virus
HCV	hepatitis C virus
TP	Treponema pallidum
TC	total cholesterol
TG	triglycerides
AE	adverse event
SAE	severe adverse event
ECG	electrocardiagram
RAPD	relative afferent pupillary defect

ULN	upper limit of normal
CRF	case report form

3. SYNOPSIS

Title	Minocycline for Retinitis Pigmentosa: a prospective, open-label, single-arm trial
Short title	Minocycline for RP
Chief investigator	Dan LIANG
Centers	This is a study involving 1 site.
Objectives	Primary Objectives:<br>To assess the efficacy of minocycline for the treatment of RP.<br>Secondary Objectives:<br>To assess safety of minocycline for the treatment of RP and the natural history of RP in Chinese population.<br>Exploratory Objectives:<br>To explore the long-term effect of minocycline for RP.
Trial/Study design	Prospective, open-label, single-arm trial.
Participants	Main eligibility criteria<br>• Participant should be in the age ranging from 18 to 60.<br>• Participant should be clinically diagnosed as RP of characteristic decreased ERG responses, retinal structure and visual field.<br>• Light-adapted 30Hz flicker ERG amplitude should not be lower than 0μV in at least one eyes of the participants.<br>• Best-corrected visual acuity (BCVA)>0.2.<br>• Participant should understand the trial and sign informed consent.<br>• Participant should comply with study procedures or follow-up visits.

	Main exclusion criteria：<br><br>• Participant is allergy to tetracycline.<br>• Participant is pregnant or lactating women.<br>• Participant is currently taking a tetracycline medication and any medication that could adversely interact with minocycline (glucocorticoids, vitamin A, DHA and others).<br>• Participant is actively receiving study therapy in another investigational study.<br>• Participants with syndromic retinitis pigmentosa including Usher Syndrome, Bardet-Biedl Syndrome.<br>• Participant is complicated with other ocular diseases including glaucoma, uveitis, aged macular degeneration (AMD), diabetic retinopathy (DR) and other ocular diseases except for age-related cataract.<br>• Participant had undergone ocular surgical interventions before except for cataract extraction.<br>• Participant has a history of uncontrolled serious concomitant illness, kidney diseases, liver diseases, autoimmune diseases, thyroid dysfunction, mental diseases or idiopathic intracranial hypertension.<br>• Participant has a history of systematic disorders which are the contraindications of examination (allergic to fluorescein) or affect the results of examination (epilepsy-like systemic neurological disease).
Study interventions	Intervention group: minocycline (100mg/day) for 24 weeks.
Duration of study	Recruitment will take place from February 2019 to September 2021.<br>Each participant will be in the study for a total of 24 weeks.
Outcome measures	Primary Outcome:<br>Change of full-field cone electroretinogram amplitude to 30Hz-flashes at week 24.<br>Secondary Outcome:<br>• Change of vision field area HFA 30-2 at week 24.<br>• Change of BCVA at week 24.

	• Change of central foveal thickness at week 24.

4. STUDY BACKGROUND

Retinitis Pigmentosa (RP) is a group of clinically and genetically heterogeneous retinal diseases characterized by progressive loss of photoreceptors and RPE. The classical features of RP include nyctalopia and progressive visual field loss. It is one of the leading causes of blindness of every age with a prevalence of 1/4000.[1, 2] Currently, there is no known effective cure for RP, which makes the patients always feel desperate, depressed and frightened for the gradually disappearing light. Interventions such as stem cell transplantation and gene therapy are challenging and still in research stage for late-stage RP patients[3, 4]. It is urgent and vital to search prospective treatments of neuro-protection that slow, stop even reverse the progression of prolong function vision in RP patients. It has been recently known that excessive oxidative stress, inflammation and apoptosis of numerous retinal cells all contribute to the progress of RP[5]. Moreover, microglia, a kind of vital immune cell located in nervous system, has been shown to counteract the production of numerous pro-inflammatory molecules and play a crucial role in the progression of RP under imbalanced oxidative and inflammatory circumstances[6, 7]. Minocycline, one of tetracycline antibiotics, has been reported to inhibit microglia activation, reduce photoreceptor apoptosis and significantly improve retinal structure and function in mouse models of RP[8]. It has been shown effective and well-tolerated in several neurodegenerative diseases such as Huntington's disease[9], multiple sclerosis[10] and Alzheimer's Disease[11]. We wonder whether minocycline can be effective and safe in RP.

5. STUDY PURPOSE AND OBJECTIVES

5.1 Primary objective

To determine the efficacy of minocycline for patients with RP by evaluating the change of visual function at week 24 visit.

5.2 Secondary objective

To determine the efficacy of minocycline for patients with RP by evaluating the change of retinal structure and mental influence at week 24 visit.

5.2.1 Key secondary objective

To evaluate the safety of minocycline for patients with RP by evaluating AEs and SAEs at week 24 visit.

5.2.2 Other secondary objective

To observe the natural history of patients with RP in Chinese population.

5.3 Exploratory objective

To explore the long-term effect of minocycline for RP.

6. STUDY DESIGN

6.1 Overview

This study is designed as a prospective, open-label, single-arm, phase 2 clinical trial. A total of thirty-five RP participants will be recruited in the study. Participants will be designed to receive minocycline (100mg) for 24 weeks and followed up for a total of 24 weeks from the date of recruitment. During this follow-up period, visits will occur at week 0, 12 and 24 of follow-up.

6.2 Scheme

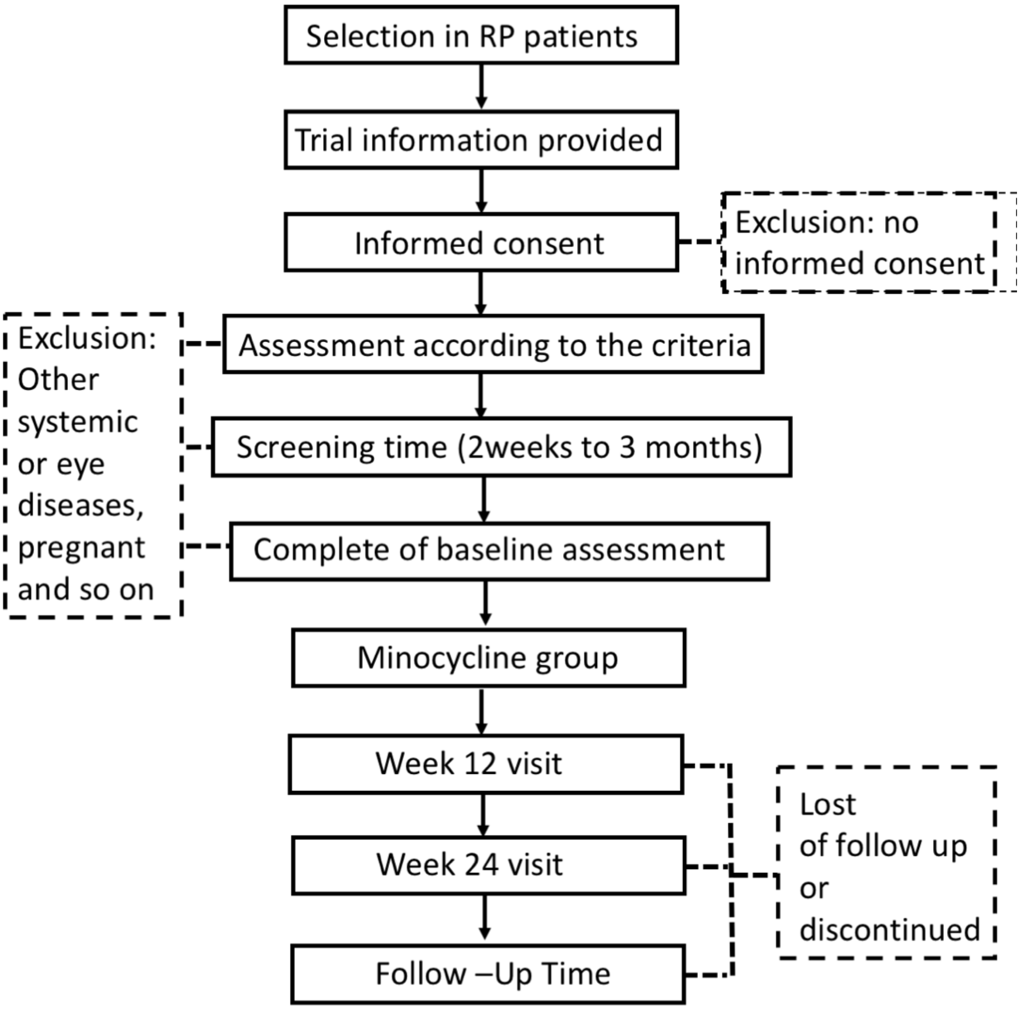

6.3 Participants adherence

To strengthen adherence of the protocol, the following strategies are applied at each visit and during the follow-up time.

- The importance of following the study guidelines for adherence is highlighted at first visit and reminded at each visit.
- During the follow-up time, for the participants are not in the hospital, a message reminder will

be sent to them.

- Instruction of investigative drugs includes dose timing, storage and numbers of capsules.
- The remaining capsules will be counted at every study visit.
- A call or a message is important if experiencing problems related to the study including symptoms, capsules lost or other condition.
- Every message, tip or reminder will be recorded as voice memos which is quite easily to be heard for the participants with poor visual acuity and visual field.

7. ADMINISTRATION OF INVESTIGATIVE DRUGS

7.1 Description

Minocycline: made in minocycline hydrochlorid capsules (100mg per capsule).

7.2 Investigative drugs making, packaging and labelling

Minocycline will be produced by Hanhui Pharmaceuticals Co., LTD. Every capsule have the same shape, volume and smell. Ten capsules make up a plate and ten plates make up a whole box. Participants will be provided a whole box of minocycline at every visit.

7.3 Storage, dispensing and return

The investigative drugs will be stored as manufacturer's instructions including protecting from sunlight and humidity. Participants will be instructed to store investigative drugs not up to 25°C.

The investigators will dispense the investigative drugs and keep detailed dispensing and returning records. All the remaining capsules and boxes will be returned by participants to the investigators. The investigators will count the remain capsules and fill in the investigative drugs record.

8. ELIGIBILITY CRITERIA

8.1 Inclusion criteria

- Participant should be in the age ranging from 18 to 60.
- Participant should be clinically diagnosed as retinitis pigmentosa with characteristic decreased ERG responses and visual fields.
- Light adapted 30Hz flicker ERG amplitude should not be lower than 0 μV in at least one eye of every participant.
- BCVA>0.2.
- Participant should understand the trial, sign informed consent and comply with study procedures or follow-up visits.
- Participant must be able to swallow pills.

- Participant must agree to minimize exposure to sunlight or artificial ultraviolet (UV) rays and to wear protective clothing, sunglasses and sunscreen [minimum sun protection factor (SPF) 15] if the participant must be out in the sun.

8.2 Exclusion criteria

- Participant is allergy to tetracycline.
- Participant is pregnant or lactating women.
- Participant is currently taking a tetracycline medication and any medication that could adversely interact with minocycline (glucocorticoids, vitamin A, DHA and others).
- Participant is actively receiving study therapy in another investigational study.
- Participant is complicated with other ocular diseases including glaucoma, uveitis, aged-macular degeneration (AMD), diabetic retinopathy (DR) and other diseases.
- Participant had undergone ocular surgical interventions.
- Participant has a history of uncontrolled serious concomitant illness, kidney diseases, liver diseases, autoimmune diseases, thyroid dysfunction, mental diseases or idiopathic intracranial hypertension.
- Participant has a history of systematic disorders which are the contraindications of examination (allergic to fluorescein) or affect the results of examination (epilepsy-like systemic neurological disease).

9. STUDY VISIT SHCEDULE AND PROCEDURE

	**Screening**	**Treatment period**		
**Study visit**	**S1/S2/S3**	**1**	**2**	**3**
**Week(W)**	**-3 to -1**	**Week 0**	**Week 12**	**Week 24**
Informed consent	X			
Review inc/exc criteria	X	X		
Demographic	X			
Height, weight	X			
Tobacco use	X			

Medical history	X			
Study drug delivery		X	X	X
Phone contact		X	X	X
**Efficacy assessments**				
BCVA	X		X	X
ERG	X		X	X
OCT	X		X	X
Perimetry	X		X	X
Fundus photography	X		X	X
FFA	X			X
**Safety assessments**				
Pregnancy test	X			
Ophthalmic exam	X	X	X	X
Mental status Evaluation	X			X
CXR	X			X
12-lead ECG electrocardiogram	X			X

Thyroid(FT3, FT4, TSH)	X			X
Blood routine	X		X	X
Urine routine	X		X	X
ALT, AST	X		X	X
BUN, Cr	X		X	X
HIV, HBV, HCV,TP	X			X
TC, TG	X			X
FBG	X			X
Concomitant medication	X		X	X
AE, SAE assessment			X	X

10. OUTCOME MEASURES

10.1 Primary outcome

Change of full-field cone electroretinogram amplitude to 30Hz-flashes at week 24.

10.2 Secondary outcome

- Change of vision field area HFA 30-2 at week 24.
- Change of BCVA at week 24.
- Change of central foveal thickness at week 24.

11. CLINICAL ASSESSMENT

11.1 General physical examination

(1) Height, Weight

Height is measured and set down as X.XX meter(m). Weight is measured and set down as XXX kilogram(kg). Weight is measured at first visit assess the baseline of participants.

(2) Blood pressure, heart rate

Participants should calm down and sit for at least 15 minutes before measuring. The same observer is supposed to use the accurate sphygmomanometer and stopwatch to measure. Additional vital signs will be monitored if associated AEs occur.

(3) Chest X Ray

Chest X Ray is measured to exclude infectious signs, for example, tuberculosis and monitor if possible associated AEs occur.

(4) Electrocardiagram (ECG)

ECG is measured to exclude cardiovascular diseases (coronary heart disease, hypertension) and monitor if possible associated AEs occur.

(5) Urine pregnancy tests

Urine pregnancy tests are performed for female patients of childbearing potential (including those with an onset of menopause <2 years prior to Screening, non-therapy-induced amenorrhea for <12 months prior to Screening, or not surgically sterile [absence of ovaries and/or uterus]).

11.2 General eye examination

(1) BCVA

BCVA will be measured using logarithm of minimal angle of resolution (LogMAR). The same observer should carry this examination according to the international standard. BCVA more than 0.0 (less than 1.0 in snellen chart) is defined as decreased vision acuity.

(2) Intraocular pressure

Intraocular pressure will first be measured using non-contact tonometer (NCT). If a rise of NCT is higher than 21mmHg, Goldman tonometer should be carried to confirm intraocular pressure. A rise in intraocular pressure is considered as abnormality which need further examination and treatment. The same observer carries this examination during the whole study. It should be set down following writing standard.

(3) Slit lamp exam

Slit lamp exam is needed to get the full screening of the eyes. If significant corneal defects, pupil abnormalities including relative afferent pupillary defect (RAPD) or others appear, further investigation of visual function will be conducted according to the ophthalmologist decision. Cause the main changes of RP are based on fundus such as optic nerve waxy pallor, bone spicule-like pigment formation and vascular damage, the eyes should be dilated by tropicamide at the first visit and the fundus should be examined carefully by the same observer to get a whole view of changes.

11.3 Lab tests

(1) Blood routine

The number of red blood cell (RBC, $*10^{12}$/L, white blood cell (WBC, $*10^{9}$/L), platelet (PLT, $*10^{9}$/L) and hemoglobin (Hb, g/L) must be within the upper limit of normal (ULN) according to age.

(2) Urine routine

Urine WBC, urine RBC, urine protein must be within ULN according to age or female period. Urine bacteria and urine fungus must be normal.

(3) Fasting blood glucose

Fasting blood glucose (mmol/L) must be within ULN.

(4) Thyroid function

Thyroid function including free triiodothyronine (FT3, pmol/L), free thyroxine (FT4, pmol/L) and thyroid stimulating hormone (TSH, mIU/L). These indexes must be within ULN.

(5) Function of lung

Alanine transaminase (ALT) /aspartate transaminase (AST) must be ≤3 x ULN according to age to be eligible.

(6) Function of kidney

Serum creatinine and blood urea nitrogen must be <1.5 x ULN according to age to be eligible.

(7) Human immunodeficiency virus (HIV), hepatitis B virus (HBV), hepatitis C virus (HCV), Treponema pallidum (TP)

They must be negative.

(8) total cholesterol (TC), triglycerides (TG)

They must be within ULN.

11.4 Efficacy

(1) ERG

Electroretinography (VerisTM, Electro-Diagnostic Imaging Inc., USA) is performed under both dark-adapted and light-adapted conditions using an International Society for Clinical Electrophysiology of Vision–compliant protocol (UTAS 3000 system; LKC Technologies). Full-field ERG responses will be performed and recorded from both eyes respectively according to ISCEV standards[12, 13]. A standard of different types of response will be measured including rod response in dark adapted eye, maximal response in dark adapted eye, cone response, response to flicker and OPS. The electronic action of retina can be recorded as a set of waveform charts characterized by implicit times and amplitudes in the ERG examination. A standard report of ERG mainly consists light adapted 30 Hz flicker ERG, light-adapted 3 ERG, dark-adapted 0.01 ERG, dark-adapted 3.0 ERG, dark-adapted 3.0 oscillatory potentials. ERG will be performed on the same machine by the particular trained performers at each visit in each participant who were blinded with the treatment and efficacy. To objectively estimate the therapeutic effect, we will enroll RP patients with recordable ERG responses in at least one eye at first visit.

(2) SD-OCT

SD-OCT images is acquired using Spectralis (Heidelberg Engineering, Heidelbery, Germany) by particular trained assessors who are masked with the treatment and efficacy of each participant. Two investigators independently manual measure the length of horizontal and vertical EZ widths. EZ widths in each eye will be measured using these cross-sectional OCT images along the horizontal and vertical meridian through the fovea.

(3) VF

VF tests are performed using Humphrey (Carl Zeiss Meditec, Dublin, California, USA) central 24–2 threshold, white-on-white automated perimetry (Swedish interactive threshold algorism fast 24–2). All VF tests are conducted in a single dark room (ambient light <5 lux). Only reliable VFs are used in the analyses, defined as a fixation loss (FL) rate of < 33% and a false-positive (FP) rate of < 33%. Unreliable fields should be repeated. VF will be performed on the same machine by the particular performers at each visit in each participant.

11.5 Safety

Safety will be assessed by evaluating adverse events (AEs). They will be examined and recorded at each visit every 3 months. Besides, a phone interview is always available for participants to contact the investigators.

(1) AE

An AE is any unfavorable and unintended sign, symptom, syndrome or illness that develops or worsens during the period of observation in the study.

An AE does include a / an:

- Exacerbation of a pre-existing illness.

- Increase in frequency or intensity of a pre-existing condition.
- Condition detected or diagnosed after study drug administration even though it may have been present prior to the start of the study.
- Continuous persistent disease or symptoms present at baseline that worsen following the start of the study.

(2) Severe Adverse Events (SAE)

A SAE is any adverse event occurring results in any of the following outcomes:

- Death
- life-threatening
- Inpatient hospitalization or prolongation of existing hospitalization.
- Persistent or significant disability/incapacity.
- A congenital anomaly or birth defect in the offspring of a participant.

Other medical events may be considered to be a SAE if they require medical or surgical intervention to prevent one of the outcomes listed in this definition.

12. DATA MANAGEMENT

12.1 Raw data forms and entry

Original raw data and forms will be kept by investigator during the study. The data should be stored in numerical order and stored in a secure and accessible place.

12.2 CRF and database

Case report form (CRF): Each participant recruited will have a CRF to record all relevant clinical data in this clinical trial. A CRF should be recorded in a timely and true manner and is supposed not be changed. When it is really necessary to correct the data, the investigator should sign and date beside the new corrected information. The CRF should also be handed over to the statistical experts, researchers and sponsors for storage after trial.

Once receiving the CRF, the data administrator should transmit the data to the investigator for verification and takes it back as soon as possible. The data administrator should establish the database in time and carry on the secondary input of the data for confirmation. After the database has been audited, the data is locked by chief investigator. To ensure the security of the data, unrelated personnel cannot access and modify the data. The data must be backed up. Any data changes need to be made with the consent of the lead investigator.

13. MANAGEMENT OF ADVERSE EVENTS

CTCAE Grade	Intensity	Definition

1	Mild	Discomfort noticed but no disruption of normal daily activity.
2	Moderate	Discomfort sufficient to reduce or affect daily activity.
3	Severe	Inability to work or perform normal daily activity.
4	Life-Threatening	Represents an immediate threat to life.
5	Fatal	Results in death.

13.1 Intensity of Adverse Events

All clinical AEs encountered during the clinical study will be reported on the AE form of the CRF. Intensity of AEs will be graded a scale of 5 points including mild, moderate, severe, life-threatening and death according to CTCAE 4.03.

13.2 Assessing relationship of adverse events to investigative drug

Causal relationship AEs to investigative drug.

DEFINITE [must have both]

This category applies to those AEs which includes laboratory test abnormality, with temporal relationship to trial treatment administration, if:

- It makes a causal relationship a reasonable possibility.
- It can definitely not be attributed to other causes. This will be counted as “related” for notification purposes.

PROBABLE [must have first three]

This category applies to those AEs which are considered, with a high degree of certainty, to be related to the test drug. An AE may be considered probable, if:

- It follows a reasonable temporal sequence from administration of the drug.
- It cannot be reasonably explained by the known characteristics of the subject’s clinical state, environmental or toxic factors, or other modes of therapy administered to the subject.
- It disappears or decreases on cessation or reduction in dose.
- It follows a known pattern of response to the suspected medication.
- It reappears upon rechallenge.

POSSIBLE [must have first two]

This category applies to those AEs in which the connection with the test drug administration appears unlikely but cannot be ruled out with certainty. An AE may be considered possible if, or when:

- It follows a reasonable temporal sequence from administration of the drug.

- It may have been produced by the subject's clinical state, environmental or toxic factors, or other modes of therapy administered to the subject.
- It follows a known pattern of response to the suspected medication.

UNLIKELY [must have first two]

In general, this category is applicable to an AE which meets the following criteria:

- It does not follow a reasonable temporal sequence from administration of the drug.
- It may readily have been produced by the subject's clinical state, environmental or toxic factors, or other modes of therapy administered to the subject.
- It does not follow a known pattern of response to the suspected medication.
- It does not reappear or worsen when the drug is re-administrated.

All AEs will be recorded and closely monitored until resolution or it has been shown that the study medication or treatment is not the cause.

13.3 Main adverse events of investigative drug

(1) Headache

The investigative drug can be dispersed through Blood-Brain Barrier and headache may occur.

(2) Gastro-intestinal discomfort

The investigative drug is required to be administrated orally daily, vomiting, nausea, diarrhea, stomachache, gastrointestinal hemorrhage, jaundice and other gastro-intestinal reaction could occur.

(3) Skin change

For the photosensitivity of tetracycline, the skin of participants may be easy to get dark if exposed to the sun too much.

13.4 Reporting of AEs

Participants will be asked to contact the study center immediately in the event of any significant adverse event. All AEs (related and unrelated) occurring during the study and up to 30 days after the last dose of study medication must be reported.

In the event of a pregnancy occurring in a trial participant or the partner of a trial participant monitoring shall occur during the pregnancy and after delivery to ascertain any trial related adverse events in the mother or the offspring.

13.5 Reporting of SAEs

A SAE will be reported immediately to the Chief Investigator and will require expedited reporting to REC.

The Chief Investigator will:

- Assess the event for seriousness, expectedness and relatedness to the investigative drug.
- Take appropriate medical action, which may include halting the trial and inform the Clinical Trial Unit and Research Ethics Committee.
- Make any amendments as required to the study protocol and inform the ethics and REC as required.

13.6 Management and follow-up of AEs and SAEs

Any AEs and SAEs present during the study will be followed until resolved or until the event stabilizes and the overall clinical outcome has been ascertained. After the study termination, AEs and SAEs will still be followed and all participants can also ask for information and medical management because of the AEs and SAEs.

In the event of unexplained, treatment-emergent, clinically significant abnormal laboratory test results or clinically significant changes in laboratory test results, the tests should be repeated immediately and followed until the values have returned to within the reference range or to baseline for that subject.

13.7 Collection of Adverse Event Data

This study is to evaluate the efficacy and safety of minocycline, which the side effect profile is well established. For this reason, only adverse events that are known as side effects will be collected.

At each visit, the investigator will identify whether the participant has experienced any of the reportable adverse events. The investigator will also have the chance to record any other essential adverse events that are not listed if deemed clinically relevant.

All available information will be collected on adverse events including hospital notes, the participant records and discussion with the participant.

14. STATICAL CONSIDERATION

Based on previous research on the natural history of retinitis pigmentosa and the stability of ERG, a sample size of 33 participants was calculated to achieve a 95% confidence interval estimate with a precision of plus or minus 15%, given an expected improvement rate of 30%.

The characteristics of the participants were presented as either mean (standard deviation) or median (interquartile range) for continuous variables, and as counts (percentage) for categorical variables. Descriptive statistics were used to assess the efficacy and safety of the intervention. The Clapper-Pearson method was used to calculate the 95% confidence interval (CI) of the proportion

parameter. The analysis of efficacy and safety included all enrolled participants who attempted minocycline treatment for RP. The statistical analysis will be performed using Stata (StataCorp).

15. ETHICAL CONSIDERATION

15.1 Ethics committee

The trial will be conducted in accordance with the ethical principles that have their origin in the Declaration of Helsinki, 1996; the principles of Good Clinical Practice, In China, a 'Guideline for Good Clinical Practice' (GCP) exists, so the investigators will strictly ensure adherence to the stated provision. The protocol will be reviewed and approved by the REC guidance and GCP with respect to scientific content and compliance with applicable research and human subject regulations.

15.2 Informed consent

Informed consent will be in accordance with the REC guidance and Good Clinical Practice (GCP). The investigators are responsible to obtain signed informed consent from each participant prior to recruiting in this study after adequate explanation of the aims, methods, anticipated benefits, and potential hazards of the study.

The original informed consents will be kept in the Investigator Site File and one copy given to the participant.

15.3 Participant welfare

The investigator must emphasize to all the participants that they are free to withdraw from the study at any time for any reason. The reason for which should be recorded. No trial-specific interventions will be done before informed consent has been obtained.

The participant will be informed of any relevant information that becomes available during the course of the study and decide whether they wish to continue with the study. If applicable they will be asked to sign revised consent forms in accordance with REC.

15.4 Criteria of discontinuing

- AE. The subject experiences an AE that imposes an unacceptable risk to the subject's health, or the subject is unwilling to continue because of an AE.
- Disease worsening. The subject experiences a progressing damage on visual function which should emergency treatment.
- Pregnancy. Subjects who prematurely discontinue study drug will return to the clinic and undergo the scheduled Week 12 assessments and will be encouraged to participate in the Follow-Up Period. Subjects who discontinue due to an AE should be followed until resolution or stabilization of the AE, or an adequate explanation for the event is obtained.
- A participant might withdraw from the study at any point for any reason, the reason for which should be recorded.

REFERENCE

1. Verbakel SK, van Huet RAC, Boon CJF, den Hollander AI, Collin RWJ, Klaver CCW, Hoyng CB, Roepman R, Klevering BJ: Non-syndromic retinitis pigmentosa. Prog Retin Eye Res 2018, 66:157-186.
2. Hartong DT, Berson EL, Dryja TP: Retinitis pigmentosa. Lancet 2006, 368(9549):1795-1809.
3. He Y, Zhang Y, Liu X, Ghazaryan E, Li Y, Xie J, Su G: Recent advances of stem cell therapy for retinitis pigmentosa. Int J Mol Sci 2014, 15(8):14456-14474.
4. Dias MF, Joo K, Kemp JA, Fialho SL, da Silva Cunha A, Jr., Woo SJ, Kwon YJ: Molecular genetics and emerging therapies for retinitis pigmentosa: Basic research and clinical perspectives. Prog Retin Eye Res 2018, 63:107-131.
5. McMurtrey JJ, Tso MOM: A review of the immunologic findings observed in retinitis pigmentosa. Surv Ophthalmol 2018, 63(6):769-781.
6. Peng B, Xiao J, Wang K, So KF, Tipoe GL, Lin B: Suppression of microglial activation is neuroprotective in a mouse model of human retinitis pigmentosa. J Neurosci 2014, 34(24):8139-8150.
7. Di Pierdomenico J, Garcia-Ayuso D, Agudo-Barriuso M, Vidal-Sanz M, Villegas-Perez MP: Role of microglial cells in photoreceptor degeneration. Neural Regen Res 2019, 14(7):1186-1190.
8. Garrido-Mesa N, Zarzuelo A, Galvez J: Minocycline: far beyond an antibiotic. Br J Pharmacol 2013, 169(2):337-352.
9. Huntington Study Group DI: A futility study of minocycline in Huntington's disease. Mov Disord 2010, 25(13):2219-2224.
10. Chen X, Ma X, Jiang Y, Pi R, Liu Y, Ma L: The prospects of minocycline in multiple sclerosis. J Neuroimmunol 2011, 235(1-2):1-8.
11. Dodel R, Spottke A, Gerhard A, Reuss A, Reinecker S, Schimke N, Trenkwalder C, Sixel-Doring F, Herting B, Kamm C et al: Minocycline 1-year therapy in multiple-system-atrophy: effect on clinical symptoms and [(11)C] (R)-PK11195 PET (MEMSA-trial). Mov Disord 2010, 25(1):97-107.
12. McCulloch DL, Marmor MF, Brigell MG, Hamilton R, Holder GE, Tzekov R, Bach M: ISCEV Standard for full-field clinical electroretinography (2015 update). Doc Ophthalmol 2015, 130(1):1-12.
13. Sustar M, Holder GE, Kremers J, Barnes CS, Lei B, Khan NW, Robson AG: ISCEV extended protocol for the photopic On-Off ERG. Doc Ophthalmol 2018, 136(3):199-206.

CLINICAL STUDY PROTOCOL

Minocycline for Retinitis Pigmentosa

Final: 6th February 2020

CONTENTS

1. STUDY PERSONNEL AND CONTACT DETAILS
2. ABBREVATIONS
3. SYNOPSIS
4. STUDY BACKGROUND
5. STUDY PURPOSE AND OBJECTIVES
    - 5.1. Primary objective
    - 5.2. Secondary objective
        - 5.2.1. Key secondary objective
        - 5.2.2. Other secondary objective
    - 5.3. Exploratory objective
6. STUDY DESIGN
    - 6.1. Overview
    - 6.2. Scheme
    - 6.3. Participants adherence
7. ADMINISTRATION OF INVESTIGATIVE DRUGS
    - 7.1. Description
    - 7.2. Investigative drugs making, packaging and labelling
    - 7.3. Storage, dispensing and return
8. ELIGIBILITY CRITERIA
    - 8.1. Inclusion criteria
    - 8.2. Exclusion criteria
9. STUDY VISIT SCHEDULE AND PROCEDURE
10. OUTCOME MEASURES
    - 10.1.Primary outcome

10.2.Secondary outcome

11. CLINICAL ASSESSMENT

11.1.General physical examination

11.2.General eye examination

11.3.Lab tests

11.4.Efficacy

11.5.Safety

12. DATA MANAGEMENT

12.1.Raw data forms and entry

12.2.CRF and database

13. MANAGEMENT OF ADVERSE EVENTS

13.1.Intensity of Adverse Events

13.2. Assessing relationship of AEs to investigative drug

13.3. Main adverse events of investigative drug

13.4. Reporting of AEs

13.5. Reporting of SAEs

13.6. Management and follow-up of AEs and SAEs

13.7. Collection of Adverse Event Data

14. STATICAL CONSIDERATION

15. ETHICAL CONSIDERATION

15.1. Ethics committee

15.2. Informed consent

15.3. Participant welfare

15.4. Criteria of discontinuing

1. STUDY PERSONNEL AND CONTACT DETAILS

Principal Investigator

Dr. Dan LIANG

Department of Ocular Immunology

Zhongshan Ophthalmic Center, Sun Yat-sen University

54 Xianlie South Road, Guangzhou, China

Tel: (+86)020-87330402

E-mail: liangdan@gzzoc.com

2. ABBREVATIONS

RP	Retinitis pigmentosa
ERG	Electroretinogram
OCT	Optical coherence tomography
VF	Visual field
CS	Contrast Sensitivity
FFA	Fluorescein angiography
CME	Cystoid macular edema
AMD	Aged-macular degeneration
DR	Diabetic retinopathy
EZ	ellipsoid zone
NCT	Non-contact tonometer
OPS	Oscillatory Potentials

MD	Mean deviation
PSD	Pattern standard deviation
HADS	Hospital Anxiety Depression Scale
NEI-VFQ-25	National Eye Institute Visual Function Questionnaire-25
RBC	red blood cell
WBC	white blood cell
PLT	platelet
Hb	hemoglobin
FT3	free triiodothyronine
FT4	free thyroxine
TSH	thyroid stimulating hormone
ALT	Alanine transaminase
AST	aspartate transaminase
HIV	Human immunodeficiency virus
HBV	hepatitis B virus
HCV	hepatitis C virus
TP	Treponema pallidum
TC	total cholesterol
TG	triglycerides
MAIA	macular integrity assessment
BCEA	bivariate contour ellipse area

FM-100	Farnsworth–Munsell 100-hue test
FACT	Functional Acuity Contrast Test
AE	adverse event
SAE	severe adverse event
CRF	case report form

3. SYNOPSIS

Title	Minocycline for Retinitis Pigmentosa: approspective, open-label, single-arm trial
Short title	Minocycline for RP
Chief investigator	Dan LIANG
Centers	This is a study involving 1 site.
Objectives	Primary Objectives:<br>To assess the efficacy of minocycline for the treatment of RP.<br>Secondary Objectives:<br>To assess safety of minocycline for the treatment of RP and the natural history of RP in Chinese population.<br>Exploratory Objectives:<br>To explore the long-term effect of minocycline for RP.
Trial/Study design	Prospective, open-label, single-arm trial.
Participants	Main eligibility criteria<br>● Participant should be in the age ranging from 18 to 60.<br>● Participant should be clinically diagnosed as RP of characteristic decreased ERG responses, retinal structure and visual field.<br>● Light-adapted 30Hz flicker ERG amplitude should not be lower than 0μV in at least one eyes of the participants.

	• Best-corrected visual acuity (BCVA) should not be less than 0 (logMAR).<br>• Participant should understand the trial and sign informed consent.<br>• Participant should comply with study procedures or follow-up visits.<br><br>Main exclusion criteria：<br><br>• Participant is allergy to tetracycline.<br>• Participant is pregnant or lactating women.<br>• Participant is currently taking a tetracycline medication and any medication that could adversely interact with minocycline.<br>• Participant is actively receiving study therapy in another investigational study.<br>• Participants with syndromic retinitis pigmentosa including Usher Syndrome, Bardet-Biedl Syndrome.<br>• Participant is complicated with other ocular diseases including glaucoma, uveitis, Aged Macular Degeneration (AMD), Diabetic Retinopathy (DR) and other ocular diseases except for age-related cataract.<br>• Participant had undergone ocular surgical interventions before except for cataract extraction.<br>• Participant has a history of uncontrolled serious concomitant illness, kidney diseases, liver diseases, autoimmune diseases, thyroid dysfunction, mental diseases or idiopathic intracranial hypertension.<br>• Participant has a history of systematic disorders which are the contraindications of examination (allergic to fluorescein) or affect the results of examination (epilepsy-like systemic neurological disease).
Study interventions	Intervention group: minocycline (100mg/day) for 12 months.
Duration of study	Recruitment will take place from February 2019 to September 2023.<br>Each participant will be in the study for a total of 12 months.

Outcome measures	Primary Outcome:<br>The proportion of participants with 10% increased amplitude of light-adapted 30Hz flicker ERG in at least one eye at month 12.<br>Secondary Outcome:<br>● The proportion of participants with increased a-wave and b-wave amplitudes in at least one eye at month 12 of the following ERG: dark-adapted 0.01 ERG, dark-adapted 3.0 ERG, dark-adapted 10.0 ERG and light-adapted 3.0 ERG.<br>● The proportion of participants with increased amplitudes of OPS in dark-adapted ERG at month 12.<br>● The proportion of participants with increased BCVA at month 12.<br>● The proportion of participants with increased mean deviation (MD) and pattern standard deviation (PSD) in Vision Field (VF) HFA 30-2 dB at month 12.<br>● The proportion of participants with increased Log CS of Contrast Sensitivity (CS) Test at month 12.<br>● The proportion of participants with decreased error scale of Color Vision at month 12.<br>● The proportion of participants with increased scores of Hospital Anxiety and Depression Scale (HADS) and National Eye Institute Visual Function Questionnaire-25 (NEI-VFQ-25) at month 12.

4. STUDY BACKGROUND

Retinitis Pigmentosa (RP) is a group of clinically and genetically heterogeneous retinal diseases characterized by progressive loss of photoreceptors and RPE. The classical features of RP include nyctalopia and progressive visual field loss. It is one of the leading causes of blindness of every age with a prevalence of 1/4000.[1, 2] Currently, there is no known effective cure for RP, which makes the patients always feel desperate, depressed and frightened for the gradually disappearing light. Intervention such as stem cell transplantation and gene therapy is challenging and still in research stage for late stage RP patients[3, 4]. It is emergency and vital to search prospective treatments of neuro-protection that slow, stop or reverse the progression of prolong function vision in RP patients.

It has been recently known that excessive oxidative stress, inflammation and apoptosis of numerous retinal cells all contribute to the progress of RP[5]. Moreover, microglia, a kind of vital immune cell located in nervous system, has been shown to counteract the production of numerous pro-inflammatory molecules and play a crucial role in the progression of RP under imbalanced

oxidative and inflammatory circumstances[6, 7]. Minocycline, one of tetracycline antibiotics, has been reported to reduce photoreceptor apoptosis, inhibit microglia activation, and significantly improve retinal structure and function in mouse models of RP[8]. It has been shown effective and well-tolerated in several neurodegenerative diseases such as Huntington's disease[9], multiple sclerosis[10] and so on[11]. We wonder whether minocycline can be effective in RP.

5. STUDY PURPOSE AND OBJECTIVES

5.1 Primary objective

To determine the efficacy of minocycline for patients with RP by evaluating the change of visual function at month 12 visit.

5.2 Secondary objective

To determine the efficacy of minocycline for patients with RP by evaluating the change of retinal structure and mental influence at month 12 visit.

5.2.1 Key secondary objective

To evaluate the safety of minocycline is safe for patients with RP by evaluating AEs and SAEs at month 12 visit.

5.2.2 Other secondary objective

To observe the natural history of patients with RP in Chinese population.

5.3 Exploratory objective

To explore the long-term effect of minocycline for RP.

6. STUDY DESIGN

6.1 Overview

This study is designed as a prospective, open-label, single-arm, phase 2 clinical trial. A total of thirty-five RP participants will be recruited in the study. Participants will be designed to receive minocycline (100mg) for 12 months and followed up for a total of 12 months from the date of recruitment. During this follow-up period, visits will occur at month 0, 3, 6, 9 and 12 of follow-up.

6.2 Scheme

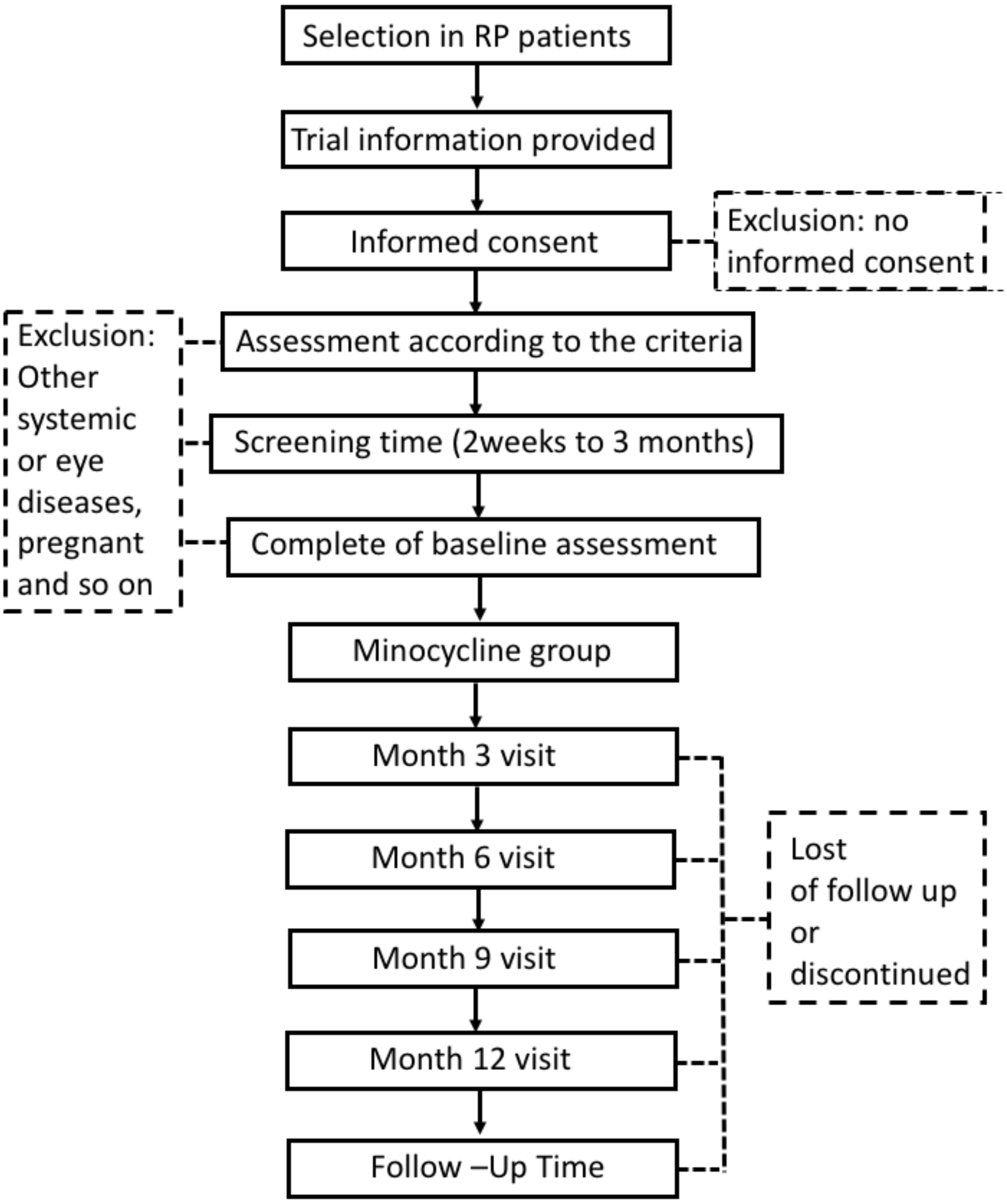

6.3 Participants adherence

To strengthen adherence of the protocol, the following strategies are applied at each visit and during the follow-up time.

- The importance of following the study guidelines for adherence is highlighted at first visit and reminded at each visit.
- During the follow-up time, for the participants are not in the hospital, a message reminder will be sent to them.
- Instruction of investigative drugs includes dose timing, storage and numbers of capsules.
- The remaining capsules will be counted at every study visit.
- A call or a message is important if experiencing problems related to the study including symptoms, capsules lost or other condition.

- Every message, tips or reminders will be recorded as voice memos which is quite easily to be heard for the participants with poor visual acuity and visual field.
- For the uncertainty of global pneumonia breakout, participants who cannot come back for follow-up should be delivered investigative drugs via express without discontinuation of drugs taking.

7. ADMINISTRATION OF INVESTIGATIVE DRUGS

7.1 Description

Minocycline: made in minocycline hydrochlorid capsules (100mg per capsule).

7.2 Investigative drugs making, packaging and labelling

Minocycline will be produced by Hanhui Pharmaceuticals Co., LTD. Every capsules have the same shape, volume and smell. Ten capsules make up a plate and ten plates make up a whole box. Participants will be provided a whole box of minocycline every visit.

7.3 Storage, dispensing and return

The investigative drugs will be stored as manufacturer's instructions including protecting from sunlight and humidity. Participants will be instructed to store investigative drugs not up to 25°C.

The investigators will dispense the investigative drugs and keep detailed dispensing and returning records. All the remaining capsules and boxes will be returned by participants to the investigators. The investigators will count the remain capsules and fill in the investigative drugs record.

8. ELIGIBILITY CRITERIA

8.1 Inclusion criteria

- Participant should be in the age ranging from 18 to 60.
- Participant should be clinically diagnosed as retinitis pigmentosa with characteristic decreased ERG responses and visual fields.
- Light adapted 30Hz flicker ERG amplitude should not be lower than 0 μV in at least one eye of every participant.
- BCVA of both eyes should not be worse than 0.
- Participant should understand the trial, sign informed consent and comply with study procedures or follow-up visits.
- Participant must be able to swallow pills.

- Participant must agree to minimize exposure to sunlight or artificial ultraviolet (UV) rays and to wear protective clothing, sunglasses and sunscreen [minimum sun protection factor (SPF) 15] if the participant must be out in the sun.

8.2 Exclusion criteria

- Participant is allergy to tetracycline.
- Participant is pregnant or lactating women.
- Participant is currently taking a tetracycline medication and any medication that could adversely interact with minocycline.
- Participant is actively receiving study therapy in another investigational study.
- Participant is complicated with other ocular diseases including glaucoma, uveitis, aged-macular degeneration (AMD), diabetic retinopathy (DR) and other diseases.
- Participant had undergone ocular surgical interventions.
- Participant has a history of uncontrolled serious concomitant illness, kidney diseases, liver diseases, autoimmune diseases, thyroid dysfunction, mental diseases or idiopathic intracranial hypertension.
- Participant has a history of systematic disorders which are the contraindications of examination (allergic to fluorescein) or affect the results of examination (epilepsy-like systemic neurological disease).

9. STUDY VISIT SHCEDULE AND PROCEDURE

	**Screening**	**Treatment period**				
**Study visit**	**S1/S2/S3**	**1**	**2**	**3**	**4**	**5**
**Month(M)**	**-3 to -1**	**Month 0**	**Month 3**	**Month 6**	**Month 9**	**Month 12**
**Visit windows （±weeks）**		**(±4)**	**(±4)**	**(±4)**	**(±4)**	**(±4)**
Informed consent	X					
Review inc/exc criteria	X	X				
Demographic	X					

Height, weight	X					
Tobacco use	X	X				
Medical history	X	X				
Study drug delivery		X	X	X	X	
Phone contact		X	X	X	X	X
**Efficacy assessments**						
BCVA	X		X	X	X	X
ERG	X		X	X	X	X
OCT	X		X	X	X	X
Perimetry	X		X	X	X	X
Microperimetry	X		X	X	X	X
Color vision	X		X	X	X	X
Contract sensitivity	X		X	X	X	X
Fundus photography	X		X	X	X	X
FFA	X			X		X
NEI-VFQ-25			X	X	X	X
HADS			X	X	X	X
**Safety assessments**						
Pregnancy test	X					
Ophthalmic exam	X	X	X	X	X	X

Mental status Evaluation	X		X	X	X	X
CXR	X			X		X
12-lead ECG electrocardiogram	X			X		X
Thyroid (FT3, FT4, TSH)	X			X		X
Blood routine	X		X	X	X	X
Urine routine	X			X		
ALT, AST	X		X	X	X	X
BUN, Cr	X		X	X	X	X
HIV, HBV, HCV,TP	X			X		X
TC, TG	X			X		X
FBG	X			X		X
Concomitant medication	X		X	X	X	
AE, SAE assessment			X	X	X	X

10. OUTCOME MEASURES

10.1 Primary outcome

The proportion of participants who get improved in light-adapted 30Hz flicker ERG amplitude at month 12. The improvement is defined as an increase not less than 10% in at least one eye.

10.2 Secondary outcome

- The proportion of participants who get improved at month 12 in a- and b- wave or OPS of other ERG amplitudes including dark-adapted 0.01 ERG, dark-adapted 3.0 ERG, dark-adapted 10.0 ERG and light-adapted 3.0 ERG. The improvement is defined as an increase not less than 10% in at least one eye.
- The proportion of participants with increased BCVA at month 12 in at least one eye.
- The proportion of participants with increased ellipsoid zone width based on OCT at month 12 in at least one eye.
- The proportion of participants with increased macular sensitivity based on microperimetry at month 12 in at least one eye.
- The proportion of participants with better VFI and MD based on 30-2 vision field measured by vision field test at month 12 in at least one eye.
- The proportion of participants with decreased error score of color vision test at month in at least one eye.
- The proportion of participants with increased contract sensitivity at month 12 in at least one eye.
- The proportion of participants with increased scores measured by NEI-VFQ-25 and HADS at month 12 in at least one eye.
- Assessment of safety duration the whole study.

11. CLINICAL ASSESSMENT

11.1 General physical examination

(1) Height, Weight

Height is measured and set down as X.XX meter(m). Weight is measured and set down as XXX kilogram(kg). Weight is measured every visit to assess the side effect of investigative drug.

(2) Blood pressure, heart rate

Participants should calm down and sit for at least 15 minutes before measuring. The same observer is supposed to use the accurate sphygmomanometer and stopwatch to measure. Additional vital signs will be monitored if associated AEs occur.

(3) Chest X Ray

Chest X Ray is measured to exclude infectious signs, for example, tuberculosis and monitor if possible associated AEs occur.

(4) Electrocardiagram (ECG)

ECG is measured to exclude cardiovascular diseases (coronary heart disease, hypertension) and monitor if possible associated AEs occur.

(5) Urine pregnancy tests

Urine pregnancy tests are performed for female patients of childbearing potential (including those with an onset of menopause <2 years prior to Screening, non-therapy-induced amenorrhea for <12 months prior to Screening, or not surgically sterile [absence of ovaries and/or uterus]).

11.2 General eye examination

(1) BCVA

BCVA will be measured using logarithm of minimal angle of resolution (LogMAR). The same observer should carry this examination according to the international standard. A loss of two lines or more is defined as damage of vision. BCVA more than 0.0 (less than 1.0 in snellen chart) is defined as decreased vision acuity.

(2) Intraocular pressure

Intraocular pressure will first be measured using non-contact tonometer (NCT). If a rise of NCT is higher than 21mmHg, Goldman tonometer should be carried to confirm intraocular pressure. A rise in intraocular pressure is considered as abnormality which need further examination and treatment. The same observer carries this examination during the whole study. It should be set down by the writing standard.

(3) Slit lamp exam

Slit lamp exam is needed to get the full screening of the eyes. If significant corneal defects, pupil abnormalities including relative afferent pupillary defect (RAPD) or others appearance, further investigation of visual function will be conducted according to the ophthalmologist decision. Cause the main changes of RP are based on fundus such as optic nerve waxy pallor, bone spicule-like pigment formation and vascular damage, the eyes should be dilated by tropicamide at the first visit and the Fundus should be examined carefully by the same observer to get a whole view of changes.

11.3 Lab tests

(1) Blood routine

The number of red blood cell (RBC, $*10^{12}/L$, white blood cell (WBC, $*10^{9}/L$), platelet (PLT, $*10^{9}/L$) and hemoglobin (Hb, g/L) must be within the upper limit of normal (ULN) according to age.

(2) Urine routine

Urine WBC, urine RBC, urine protein must be within ULN according to age or female period. Urine bacteria and urine fungus must be normal.

(3) Fasting blood glucose

Fasting blood glucose (mmol/L) must be within ULN.

(4) Thyroid function

Thyroid function including free triiodothyronine (FT3, pmol/L), free thyroxine (FT4, pmol/L) and thyroid stimulating hormone (TSH, mIU/L) and must be within ULN.

(5) Function of lung

Alanine transaminase (ALT) /aspartate transaminase (AST) must be ≤3 x ULN according to age to be eligible.

(6) Function of kidney

Serum creatinine and blood urea nitrogen must be <1.5 x ULN according to age to be eligible.

(7) Human immunodeficiency virus (HIV), hepatitis B virus (HBV), hepatitis C virus (HCV), Treponema pallidum (TP)

They must be negative.

(8) total cholesterol (TC), triglycerides (TG)

They must be within ULN.

11.4 Efficacy

(1) ERG

Electroretinography (VerisTM, Electro-Diagnostic Imaging Inc., USA) is performed under both dark-adapted and light-adapted conditions using an International Society for Clinical Electrophysiology of Vision–compliant protocol (UTAS 3000 system; LKC Technologies). Full-field ERG responses will be performed and recorded from both eyes respectively according to ISCEV standards[12, 13]. A standard of different types of response will be measured including rod response in dark adapted eye, maximal response in dark adapted eye, cone response, response to flicker and OPS. The electronic action of retina can be recorded as a set of waveform charts characterized by implicit times and amplitudes in the ERG examination. A standard report of ERG mainly consists light adapted 30 Hz flicker ERG, light-adapted 3 ERG, dark-adapted 0.01 ERG,

dark-adapted 3.0 ERG, dark-adapted 3.0 oscillatory potentials. ERG will be performed on the same machine by the particular trained performers at each visit in each participant who were blinded with the treatment and efficacy. To objectively estimate the therapeutic effect, we will enroll RP patients with recordable ERG responses in at least one eye at first visit.

(2) SD-OCT

SD-OCT images is acquired using Spectralis (Heidelberg Engineering, Heidelbery, Germany) by particular trained assessors who are masked with the treatment and efficacy of each participant. Two investigators independently manual measure the length of horizontal and vertical EZ widths. EZ widths in each eye will be measured using these cross-sectional OCT images along the horizontal and vertical meridian through the fovea.

(3) VF

VF tests are performed using Humphrey (Carl Zeiss Meditec, Dublin, California, USA) central 24–2 threshold, white-on-white automated perimetry (Swedish interactive threshold algorism fast 24–2). All VF tests are conducted in a single dark room (ambient light <5 lux). Only reliable VFs are used in the analyses, defined as a fixation loss (FL) rate of < 33% and a false-positive (FP) rate of < 33%. Unreliable fields should be repeated. VF will be performed on the same machine by the particular performers at each visit in each participant.

(4) Microperimetry

Microperimetry is performed using a macular integrity assessment (MAIA) by an expert 4-2 examination covering 10 degrees of diameter of the macular area. The examination includes 37 measurement points in three circles with 2, 6, and 10 degrees of diameter, respectively. The stimulus is Goldman III in magnitude and lasts 200ms. The illumination of the stimulus is distributed from 0 to 36 dB. The results of average threshold of macular sensitivity, macular integrity index, P1, P2, 63% bivariate contour ellipse area (BCEA), and 95% BCEA will be analyzed and classified and it will be performed on the same machine by the particular performers at each visit in each participant.

(5) Color Vision

Color Vision is measured using Farnsworth–Munsell 100-hue test (FM-100) (Munsell Color Company, Inc., Baltimore, MD, USA) under illuminance of 100Lux[14]. Participants are asked to arrange 85 colored disks in four racks of 20 to 22 disks, in a sequence with a gradual progression of hue between adjacent disks. Both eyes will be measured respectively with a viewing distance of 0.5m under BCVA and the time for every rack should be limited to 2 minutes. Error scores for each eye will be evaluated and set down by the particular performer at each visit in each participant.

(6) CS

CS is measured using the Functional Acuity Contrast Test (FACT) chart (Stereo Optical, Inc., Chicago, IL, USA) with room illumination at 85 cd/m2[15]. The FACT chart consists of a series of 45 sine wave gratings corresponding to nine levels of CS at five spatial frequencies. The

participants are tested monocularly at each spatial frequency in a random sequence with a viewing distance of 0.5m under BCVA. LogCS values will be used for analysis by the particular performer at each visit in each participant.

(7) Quality of life (QoL)

Quality of life is assessed at enrollment and each visit using the NEI-VFQ-25, consisting of 25 vision-targeted questions and representing 11 vision-related constructs and a general health rating question (general health, general vision, ocular pain, near activities, distance activities, social functioning, mental health, role difficulties, dependency, color vision, peripheral vision, and driving).[16] All items are scored on a 0 to 100 scale with a high score representing better functioning. An overall Composite Score will be calculated as the average of the vision-targeted subscale scores, excluding the General Health rating question. For each subscale, the changes from baseline is calculated in terms of the average score increase/decrease, and as the average of percentage changes in scores. Participants will be provided with questionnaire instructions to complete on their own. The same investigator is available to clarify wording, but otherwise provided no feedback or assistance with completing the questionnaire.

(8) Anxiety and depression

HADS is applied to judge participants' condition of anxiety and depression. It consists of 14-item questions which can be divided into two 7-item subscales named HADS-A and HADS-D. Each question has different point scale ranging from 0 to 3[17]. A higher score means a worse condition of anxiety and depression. The subscales made up of each question will be recorded by the same investigator.

11.5 Safety

Safety will be assessed by evaluating adverse events (AEs). They will be examined and recorded at each visit every 3 months. Besides, a phone interview is always available for participants to contact the investigators.

(1) AE

An AE is any unfavorable and unintended sign, symptom, syndrome or illness that develops or worsens during the period of observation in the study.

An AE does include a / an:

- Exacerbation of a pre-existing illness.
- Increase in frequency or intensity of a pre-existing condition.
- Condition detected or diagnosed after study drug administration even though it may have been present prior to the start of the study.
- Continuous persistent disease or symptoms present at baseline that worsen following the start of the study.

(2) Severe Adverse Events (SAE)

A SAE is any adverse event occurring results in any of the following outcomes:

- Death

- life-threatening
- Inpatient hospitalization or prolongation of existing hospitalization.
- Persistent or significant disability/incapacity.
- A congenital anomaly or birth defect in the offspring of a participant.

Other medical events may be considered to be a SAE if they require medical or surgical intervention to prevent one of the outcomes listed in this definition.

12. DATA MANAGEMENT

12.1 Raw data forms and entry

Original raw data and forms will be kept by investigator during the study. The data should be stored in numerical order and stored in a secure and accessible place.

12.2 CRF and database

Case report form (CRF): Each participant recruited will have a CRF to record all relevant clinical data in this clinical trial. A CRF should be recorded in a timely and true manner and is supposed not be changed. When it is really necessary to correct the data, the investigator should sign and date beside the new corrected information. The CRF should also be handed over to the statistical experts, researchers and sponsors for storage after trial.

Once receiving the CRF, the data administrator should transmit the data to the investigator for verification and takes it back as soon as possible. The data administrator should establish the database in time and carry on the secondary input of the data for confirmation. After the database has been audited, the data is locked by chief investigator. To ensure the security of the data, unrelated personnel cannot access and modify the data. The data must be backed up. Any data changes need to be made with the consent of the lead investigator.

13. MANAGEMENT OF ADVERSE EVENTS

CTCAE Grade	Intensity	Definition
1	Mild	Discomfort noticed but no disruption of normal daily activity.
2	Moderate	Discomfort sufficient to reduce or affect daily activity.
3	Severe	Inability to work or perform normal daily activity.
4	Life-Threatening	Represents an immediate threat to life.

5	Fatal	Results in death.

13.1 Intensity of Adverse Events

All clinical AEs encountered during the clinical study will be reported on the AE form of the CRF. Intensity of AEs will be graded a scale of 5 points including mild, moderate, severe, life-threatening and death according to CTCAE 4.03.

13.2Assessing relationship of adverse events to investigative drug

Causal relationship AEs to investigative drug.

DEFINITE [must have both]

This category applies to those AEs which includes laboratory test abnormality, with temporal relationship to trial treatment administration, if:

- It makes a causal relationship a reasonable possibility.
- It can definitely not be attributed to other causes. This will be counted as “related” for notification purposes.

PROBABLE [must have first three]

This category applies to those AEs which are considered, with a high degree of certainty, to be related to the test drug. An AE may be considered probable, if:

- It follows a reasonable temporal sequence from administration of the drug.
- It cannot be reasonably explained by the known characteristics of the subject’s clinical state, environmental or toxic factors, or other modes of therapy administered to the subject.
- It disappears or decreases on cessation or reduction in dose.
- It follows a known pattern of response to the suspected medication.
- It reappears upon rechallenge.

POSSIBLE [must have first two]

This category applies to those AEs in which the connection with the test drug administration appears unlikely but cannot be ruled out with certainty. An AE may be considered possible if, or when:

- It follows a reasonable temporal sequence from administration of the drug.
- It may have been produced by the subject’s clinical state, environmental or toxic factors, or other modes of therapy administered to the subject.
- It follows a known pattern of response to the suspected medication.

UNLIKELY [must have first two]

In general, this category is applicable to an AE which meets the following criteria:

- It does not follow a reasonable temporal sequence from administration of the drug.
- It may readily have been produced by the subject’s clinical state, environmental or toxic factors, or other modes of therapy administered to the subject.

- It does not follow a known pattern of response to the suspected medication.
- It does not reappear or worsen when the drug is readministrated.

All AEs will be recorded and closely monitored until resolution or it has been shown that the study medication or treatment is not the cause.

13.3 Main adverse events of investigative drug

(1) Headache

The investigative drug can be dispersed through Blood-Brain Barrier and headache may occur.

(2) Gastro-intestinal discomfort

The investigative drug is required to be administrated orally daily, vomiting, nausea, diarrhea, stomachache, gastrointestinal hemorrhage, jaundice and other gastro-intestinal reaction could occur.

(3) Skin change

For the photosensitivity of tetracycline, the skin of participants may be easy to get dark if exposed to the sun too much.

13.4 Reporting of AEs

Participants will be asked to contact the study center immediately in the event of any significant adverse event. All AEs (related and unrelated) occurring during the study and up to 30 days after the last dose of study medication must be reported.

In the event of a pregnancy occurring in a trial participant or the partner of a trial participant monitoring shall occur during the pregnancy and after delivery to ascertain any trial related adverse events in the mother or the offspring.

13.5 Reporting of SAEs

A SAE will be reported immediately to the Chief Investigator and will require expedited reporting to REC.

The Chief Investigator will:

- Assess the event for seriousness, expectedness and relatedness to the investigative drug.
- Take appropriate medical action, which may include halting the trial and inform the Clinical Trial Unit and Research Ethics Committee.
- Make any amendments as required to the study protocol and inform the ethics and REC as

required.

13.6 Management and follow-up of AEs and SAEs

Any AEs and SAEs present during the study will be followed until resolved or until the event stabilizes and the overall clinical outcome has been ascertained. After the study termination, AEs and SAEs will still be followed and all participants can also ask for information and medical management because of the AEs and SAEs.

In the event of unexplained, treatment-emergent, clinically significant abnormal laboratory test results or clinically significant changes in laboratory test results, the tests should be repeated immediately and followed until the values have returned to within the reference range or to baseline for that subject.

13.7 Collection of Adverse Event Data

This study is to evaluate the efficacy and safety of minocycline, which the side effect profile is well established. For this reason, only adverse events that are known as side effects will be collected.

At each visit, the investigator will identify whether the participant has experienced any of the reportable adverse events. The investigator will also have the chance to record any other essential adverse events that are not listed if deemed clinically relevant.

All available information will be collected on adverse events including hospital notes, the participant records and discussion with the participant.

14 STATICAL CONSIDERATION

Based on previous research on the natural history of retinitis pigmentosa and the stability of ERG, a sample size of 33 participants was calculated to achieve a 95% confidence interval estimate with a precision of plus or minus 15%, given an expected improvement rate of 30%.

The characteristics of the participants were presented as either mean (standard deviation) or median (interquartile range) for continuous variables, and as counts (percentage) for categorical variables. Descriptive statistics were used to assess the efficacy and safety of the intervention. The Clapper-Pearson method was used to calculate the 95% confidence interval (CI) of the proportion parameter. The analysis of efficacy and safety included all enrolled participants who attempted minocycline treatment for RP. The statistical analysis will be performed using Stata(StataCorp).

15 ETHICAL CONSIDERATION

15.1 Ethics committee

The trial will be conducted in accordance with the ethical principles that have their origin in the Declaration of Helsinki, 1996; the principles of Good Clinical Practice, In China, a 'Guideline for Good Clinical Practice' (GCP) exists, so the investigators will strictly ensure adherence to the

stated provision. The protocol will be reviewed and approved by the REC guidance and GCP with respect to scientific content and compliance with applicable research and human subject regulations.

15.2 Informed consent

Informed consent will be in accordance with the REC guidance and Good Clinical Practice (GCP). The investigators are responsible to obtain signed informed consent from each participant prior to recruiting in this study after adequate explanation of the aims, methods, anticipated benefits, and potential hazards of the study.

The original informed consents will be kept in the Investigator Site File and one copy given to the participant.

15.3 Participant welfare

The investigator must emphasize to all the participants that they are free to withdraw from the study at any time for any reason. The reason for which should be recorded. No trial-specific interventions will be done before informed consent has been obtained.

The participant will be informed of any relevant information that becomes available during the course of the study and decide whether they wish to continue with the study. If applicable they will be asked to sign revised consent forms in accordance with REC.

15.4 Criteria of discontinuing

- AE. The subject experiences an AE that imposes an unacceptable risk to the subject's health, or the subject is unwilling to continue because of an AE.
- Disease worsening. The subject experiences a progressing damage on visual function which should emergency treatment.
- Pregnancy. Subjects who prematurely discontinue study drug will return to the clinic and undergo the scheduled Week 12 assessments and will be encouraged to participate in the Follow-Up Period. Subjects who discontinue due to an AE should be followed until resolution or stabilization of the AE, or an adequate explanation for the event is obtained.
- A participant might withdraw from the study at any point for any reason, the reason for which should be recorded.

REFERENCE

1. Verbakel SK, van Huet RAC, Boon CJF, den Hollander AI, Collin RWJ, Klaver CCW, Hoyng CB, Roepman R, Klevering BJ: **Non-syndromic retinitis pigmentosa**. *Prog Retin Eye Res* 2018, **66**:157-186.
2. Hartong DT, Berson EL, Dryja TP: **Retinitis pigmentosa**. *Lancet* 2006, **368**(9549):1795-1809.
3. He Y, Zhang Y, Liu X, Ghazaryan E, Li Y, Xie J, Su G: **Recent advances of stem cell therapy for retinitis pigmentosa**. *Int J Mol Sci* 2014, **15**(8):14456-14474.
4. Dias MF, Joo K, Kemp JA, Fialho SL, da Silva Cunha A, Jr., Woo SJ, Kwon YJ: **Molecular genetics and emerging therapies for retinitis pigmentosa: Basic research and clinical perspectives**. *Prog Retin Eye Res* 2018, **63**:107-131.
5. McMurtrey JJ, Tso MOM: **A review of the immunologic findings observed in retinitis pigmentosa**. *Surv Ophthalmol* 2018, **63**(6):769-781.
6. Peng B, Xiao J, Wang K, So KF, Tipoe GL, Lin B: **Suppression of microglial activation is neuroprotective in a mouse model of human retinitis pigmentosa**. *J Neurosci* 2014, **34**(24):8139-8150.
7. Di Pierdomenico J, Garcia-Ayuso D, Agudo-Barriuso M, Vidal-Sanz M, Villegas-Perez MP: **Role of microglial cells in photoreceptor degeneration**. *Neural Regen Res* 2019, **14**(7):1186-1190.
8. Garrido-Mesa N, Zarzuelo A, Galvez J: **Minocycline: far beyond an antibiotic**. *Br J Pharmacol* 2013, **169**(2):337-352.
9. Huntington Study Group DI: **A futility study of minocycline in Huntington's disease**. *Mov Disord* 2010, **25**(13):2219-2224.
10. Chen X, Ma X, Jiang Y, Pi R, Liu Y, Ma L: **The prospects of minocycline in multiple sclerosis**. *J Neuroimmunol* 2011, **235**(1-2):1-8.
11. Dodel R, Spottke A, Gerhard A, Reuss A, Reinecker S, Schimke N, Trenkwalder C, Sixel-Doring F, Herting B, Kamm C *et al*: **Minocycline 1-year therapy in multiple-system-atrophy: effect on clinical symptoms and [(11)C] (R)-PK11195 PET (MEMSA-trial)**. *Mov Disord* 2010, **25**(1):97-107.
12. McCulloch DL, Marmor MF, Brigell MG, Hamilton R, Holder GE, Tzekov R, Bach M: **ISCEV Standard for full-field clinical electroretinography (2015 update)**. *Doc Ophthalmol* 2015, **130**(1):1-12.
13. Sustar M, Holder GE, Kremers J, Barnes CS, Lei B, Khan NW, Robson AG: **ISCEV extended protocol for the photopic On-Off ERG**. *Doc Ophthalmol* 2018, **136**(3):199-206.
14. Kinnear PR: **Proposals for scoring and assessing the 100-Hue test** *Vision Res* 1970, **10**(5):423-433.
15. K Pesudovs 1 CAH, R M L Doran, D B Elliott: **The usefulness of Vistech and FACT contrast sensitivity charts for cataract and refractive surgery outcomes research**. *Br J Ophthalmol* 2004, **88**(1):11-16.
16. Toprak AB, Eser E, Guler C, Baser FE, Mayali H: **Cross-validation of the Turkish version of the 25-item National Eye Institute Visual Functioning Questionnaire (NEI-VFQ 25)**. *Ophthalmic Epidemiol* 2005, **12**(4):259-269.
17. A S Zigmond RPS: **The hospital anxiety and depression scale**. *Acta Psychiatr Scand* 1983, **67**(6):361-370.

Summary of changes

1. For a number of uncertain situations of the global pneumonia outbreak, we changed the follow-up time from 24 weeks to 12 months. Also, a delivery of investigative drugs via post is permitted if any participants have difficulty in coming back for follow-up examination.
2. We changed the primary and secondary outcome of changes in ERG, BCVA and other indexes to the proportion of participants who get improved in light-adapted 30Hz flicker ERG amplitude at month 12. The improvement is defined as an increase not less than 10% in at least one eye.
3. We changed the inclusion criteria of BCVA >0.2 into better than zero, for the difficulty of enrollment of suitable RP participants.
